# Solid-state Reaction of Azolium Hydrohalogen Salts with Silver Dicyanamide – Unexpected Formation of Cyanoguanidine-azoles, Reaction Mechanism and Their Hypergolic Properties

**DOI:** 10.1038/srep10915

**Published:** 2015-06-03

**Authors:** Wei Liu, Qiu-han Lin, Yu-chuan Li, Peng-wan Chen, Tao Fang, Ru-bo Zhang, Si-ping Pang

**Affiliations:** 1 School of Materials Science and Engineering, Beijing Institute of Technology, Beijing, 100081, China; 2 School of Chemical Engineering and Environment, Nanjing Institute of Technology, Nanjing, 210094, China; 3 Academy of Aerospace Propulsion Technology, Beijing, China; 4 School of Chemistry, Beijing Institute of Technology, Beijing, 100081, China; 5 State Key Laboratory of Explosion Science and Technology, Beijing Institute of Technology, Beijing, 100081, China; 6 Beijing Centre For Physical and Chemical Analysis, Beijing, 100089, China

## Abstract

Cyanoguanidines as well as azoles are important bioactive groups, which play an important role in the medical application; meanwhile, the high nitrogen content makes them excellent backbones for energetic materials. A Novel and simple method that combined these two fragments into one molecular compound was developed through the transformation of dicyanamide ionic salts. In return, compounds **4**–**11** were synthesized, and fully characterized by IR, MS, NMR and elemental analysis. Meanwhile, the structures of compounds **4**, **8** and **11** were confirmed by X-ray crystal diffraction. Detailed reaction mechanisms were studied through accurate calculations on the reaction energy profiles of the azolium cations and DCA anion, which revealed the essence of the transformation proceeding. Meanwhile, compound **8** exhibits excellent hypergolic property, which could be potentially novel molecular hypergolic fuel.

Cyanoguanidines, as important derivatives of nitriles, are polyfunctional species containing both the cyano and guanidine groups. Derivatives of cyanoguanidine have been found a number of applications in the fields such as high energetic materials^1^,^2^,^3^, medicine^4^ and environmental protection^5^.

For example, cyanoguanidine derivatives of pyridazinones have potent antisecretory and/or antiulcer activities^6^, while derivatives of loratadine have potential antitumor activity^7^. In addition, cyanoguanidines are important intermediates since the cyano group can be easily converted into a variety of functional groups^8^,^9^,^10^,^11^, such as it is a precursor for the synthesis of some organonitrogen compounds.

Furthermore, the cyanoguanidine group has strong affinity interaction with mercury, and thus prominent adsorption capacity for Hg in aqueous phase with extraordinary selectivity, which has been adopted to remedy for heavy metal pollution. Although methods of cyanation have been extensively studied^12^,^13^,^14^,^15^, synthetic methodologies for preparation of cyanoguanidine are limited. For example, the cyanoguanidine functional group could be prepared from thiorea and mostly involved the isolation of intermediate of carbodiimide^16^.

Azoles are also important bioactive groups, which are widely used in the synthesis of medicine. Meanwhile, due to their high nitrogen content, azoles were often used to construct the polynitrogen energetic compounds^17^,^18^. Recently, dicyanamide anion-containing ionic liquids (DCA-ILs) received a considerable attention for their hypergolic properties^19^,^20^,^21^,^22^,^23^. However, the used cations were limited to the alkyl substituted imidazoles or hyamines. In our attempts to prepare more energetic 1,2,3-triazolium and 1-amino-3-methyl-1,2,3-triazolium dicyanamide salts, the targeted ionic salts were not obtained, but molecular compounds containing both azole and cyanoguanidine groups were obtained instead. The simple combination of azoles and cyanoguanidine into one compound attracted our interests strongly. Though Cu^2+^-mediated nucleophilic addition of pyrazoles to dicyanamide was reported^24^,^25^, to the best of our knowledge, the transformation between the heterocyclic cations and DCA anion in solid state is unprecedented.

To confirm whether this transformation could occur between DCA anion and other heterocyclic cations, we explored a series of azole hydrochlorides as substrates, including imidazolium, pyrazolium, 1,2,4-triazolium, and their amino derivatives, 4-amino-1,2,4-triazolium, 1-amino-1,2,3-triazolium and 3,5-diamino-1,2,4-triazolium hydrochlorides (Fig. 1). Proposed mechanism of our new chemical reaction was studied by using DFT calculations.

## Results

**Figure 1:**
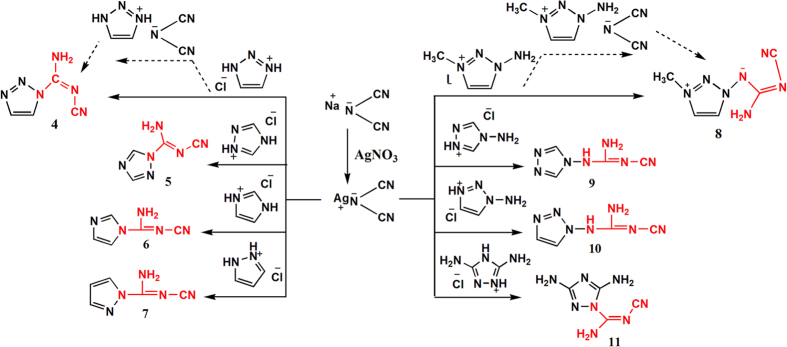
Reactions of silver dicyanamide with various azolium salts.

The synthesis of cyanoguanidine-azoles involved a simple two-step reaction, initiated by ion-exchange between azolium chloride or iodide with silver dicyanamide (resulted in a precipitation of AgCl or AgI), in aqueous solution. The second step included filtration of the silver salt, evaporation of the resulted solution with subsequent solid-state reaction within the residues, to yield target high-nitrogen content products **4**-**11** (Fig. 1). Formation of some these products, required a thermal treatment, in order to promote better yields of the desired compounds.

All products were isolated and characterized by IR, ^1^H NMR, ^13^C NMR, MS spectrum and elemental analysis. The structures of compounds **4**, **8** and **11** were confirmed by X-ray crystallography.

MS spectrum is a direct confirmation of the transformation. The mass peaks of the transformation molecular products would appear in the spectrum if the transformation reactions happened. Otherwise, only mass peaks of the heterocyclic cations would appear. For example, taking imidazolium cation and DCA anion, the signal at m/z 134 could be assigned to the transformation product **6**, which appears in ESI-MS spectrum, stating that the ionic complex was transformed into the compound in the solid. Analogous phenomena could be observed in corresponding ESI-MS for each of compounds of **4**-**11**.

**Figure 2:**
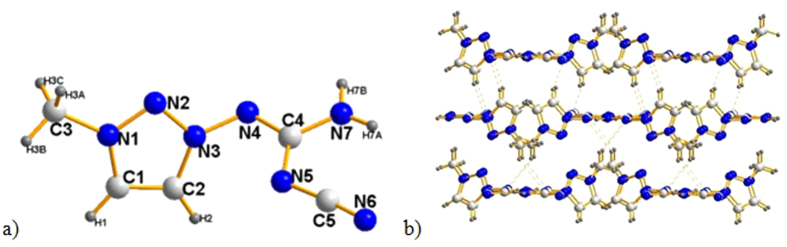
(**a**) Diamond representation of the molecular structure of **8**. Displacement ellipsoids are shown at 50% probability level. (**b**) Unit cell packing of **8**. Blue spheres represent nitrogen atoms, gray spheres represent carbon atoms, and smaller gray spheres represent hydrogen atoms.

Crystals of compounds **4**, **8** and **11,** suitable for X-ray crystallography were obtained from methonal. For compound **4**, the N2–N3–N4–C4, C2–N3–N4–C4 dihedral angles are 119.11(145)° and −70.58(195)°, respectively, indicating that the 3-cyanoguanidino group exhibits a considerable bending from the plane of a triazole ring, whereas the triazole ring and methyl group are coplanar [torsion angle C2–C1–N1–C3 177.09(147)°]. Also, the triazole rings and 2-cyanoamidino group [torsion angle N2–N3–C3–N5 –174.56(25)°, N2–N3–C3–N4 5.61(409)°] are coplanar in compound **8** (Fig. 2). The two amino groups in **11** lay perfectly within the plane of the 1,2,4-triazole ring. However, the hydrogen atoms of the amino groups in **11** are significantly twisted out of the plane of the triazole ring, with the maximum torsion angle of –29.5°. Moreover, we observed that, the incorporation of 2-cyanoamidino into the triazole ring results in a lengthening of the C1–N1 bond (1.386 Å) and N1–N2 bond (1.409 Å). The 2-cyanoamidino group lays in the plane of the triazole ring, as clearly shown by the N1–C3–N6–H6B torsion angle of –175.3° and N1–C3–N7–C4 torsion angle of –179.5°. Details of crystal data, bond lengths and angles are presented in Tables 1, 2 and 3.

## Discussion

**Figure 3:**
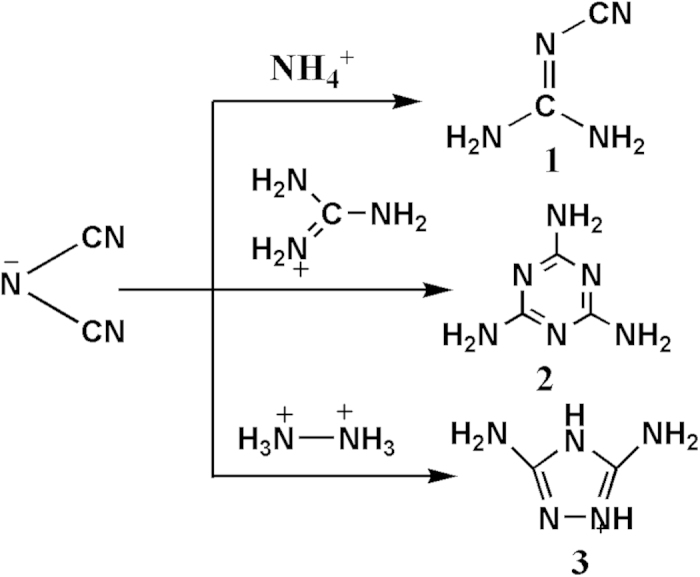
Reported transformations of dicyanamide anion.

Why treatment of the azolium salts with the DCA could be transformed to the molecular products? Actually, the ionic salts between dicyanamide and ammonium^26^,^27^, guanidinium^28^, hydrazinium^29^ were successfully converted into cyano-guanidine, melamine and 3,5-diamino-1,2,4-triazole, respectively (Fig. 3). The relevant reaction mechanisms were also proposed, but not further confirmed. To better understand the transformation reaction mechanism, we carried on the theoretical studies in details.

All the geometries were optimized by M06-2X/6-31+G(d,p) method^30^ without any constraint. Frequencies were calculated to ascertain the local minimum or transition structures. Single-point energies were calculated with the same functional together with higher aug-cc-pVTZ basis set. The combination could give the very accurate reaction barrier and energies of polynitrogen molecules, which can well reproduce the benchmark values obtained at the CCSD(T)/aug-cc-pVTZ level^31^. The implicit C-PCM solvation model^32^ was used to simulate the aqueous surroundings in the single-point energy calculations. All the calculations were implemented with G09 suits^33^.

**Figure 4:**
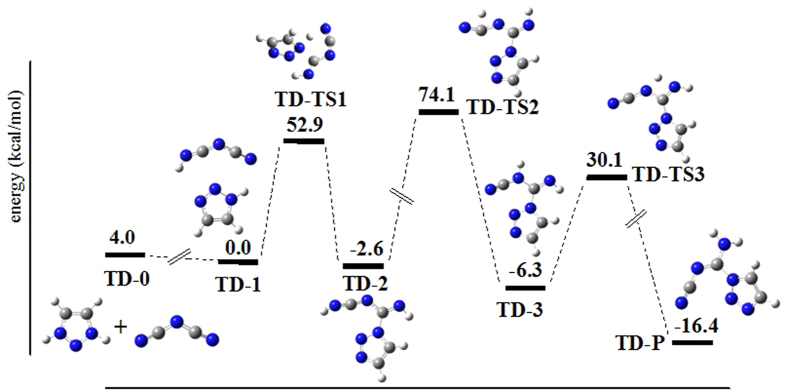
Theoretical calculation of reaction pathway for the transformation of 1,2,3-triazolium cation and dicyanamide anion to product 4. Blue spheres represent nitrogen atoms, gray spheres represent carbon atoms, and white spheres represent hydrogen atoms.

For unsubstituted azolium cations such as 1,2,3-triazolium DCA in aqueous solution, our calculations show that the protonated 1,2,3-triazolium could form the hydrogen-bonding complex TD-1 when mixed with DCA anion through exothermic heat of only 4.0 kcal·mol^−1^. During TD-1 structure optimization, one proton could move to DCA anion with no barrier, which results in TD-1 formation. For TD-1 complex, the favoring interaction energy is estimated to be 3.7 kcal mol^−1^. The calculation indicates that TD-1 could be in aqueous solution although it is quite weak. The subsequent N-C bond is formed upon attack of γ-nitrogen atom to the carbon atom of DCA. Consistently, the other proton attached to the above nitrogen atom could be transferred to the terminal nitrogen atom of DCA anion (Fig. 4). Through the two successive proton transfer reactions with *quite high* barrier heights, the product TD-P (compound **4**) could be available. Starting from the reactive complex TD-1 to the final product, the net reaction is exothermal by ca. 16.4 kcal·mol^−1^.

For 1-amino-3-methyl-1,2,3-triazolium cation, the reaction mechanism should be a little different from that of the unsubstituted azolium cations. The dissociated proton can be provided by only the exocyclic NH_2_ group, which is clearly distinct from the case in the 1,2,3-triazolium cation.

**Figure 5:**
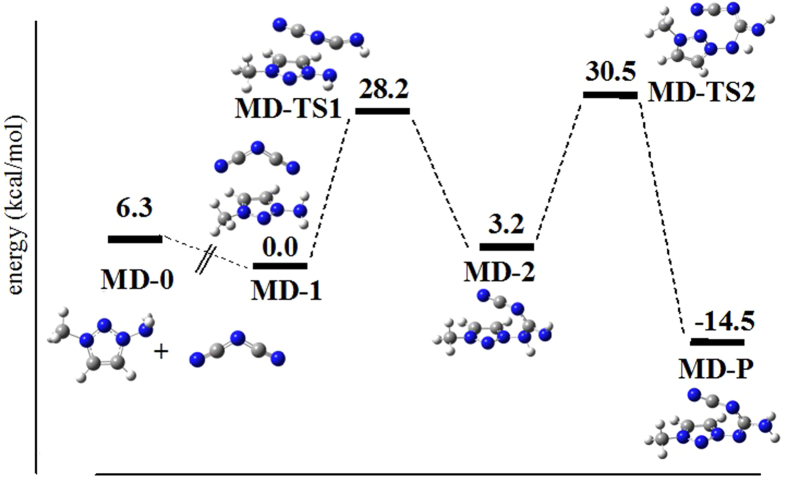
Theoretical calculation of reaction pathway for the transformation of 3-methyl-1-amino-1,2,3-triazolium cation and dicyanamide anion into compound **8**. Blue spheres represent nitrogen atoms, gray spheres represent carbon atoms, and white spheres represent hydrogen atoms.

Thus, the stable reactive complex MD-1 could be formed through exothermic heat of ca. 6.3 kcal·mol^−1^ (Fig. 5), which is comparable to the corresponding value of TD-1 formation. The trivial difference should be attributed to the fact that the protonated azolium ring has stronger acidity than 1-amino-3-methyl-1,2,3-triazolium. Their acidities can be assessed by the calculations of their deprotonated enthalpies (DPE) at the same theoretical level. Their gaseous DPEs are 261.2 kcal·mol^−1^ for 1,2,3-triazolium cation and 286.9 kcal·mol^−1^ for 1-amino-3-methyl-1,2,3-triazolium cation, respectively. The subsequent cross-link product is formed upon attack of the same nitrogen atom in the exocyclic amino group to the carbon of DCA, which is different from that of 1,2,3-triazolium. Consistently, one proton in the NH_2_ group could be transferred to the terminal nitrogen atom in DCA. The reaction is trivially endothermic and has a *moderate* barrier (28.2 kcal·mol^−1^) to be overcome to reach the corresponding intermediate MD-2, from which the other proton transferred to -NH of DCA gives the product MD-P (compound **8**).

Note that both compounds 4 (that is, TD-P in Fig. 4) and 8 (that is, MD-P in Fig. 5) have the same reaction temperature and the similar yield. The reaction time difference, however, is quite large as seen in Table 4. For the primary cross-linked reactions, the endothermic heat is 48.9 for TD-0 → TD-2 and 21.9 kcal mol^−1^ for MD-0 → MD-2, which are calculated relative to the two isolated molecules. Thus, formation of TD-2 should have the reaction time more than formation of MD-2 does. For the subsequent reactions towards the final product, formation of TD-P needs to successively override two barriers of 76.7 and 36.4 kcal mol^−1^. These data are clearly higher than one barrier of 27.3 kcal mol^−1^ for MD-P formation, seen in Figs 4 and 5. Combined with the present experimental results, the total reaction time should depend on the total reaction paths.

Other cations’ reactions with DCA anion share the similar mechanism. (seen in Supporting Information) The present calculations also discover the reaction essence of the azolium cation with DCA anion. The proton transfer is necessary for the subsequent reactions. Acidity of the proton in azolium rings is normally stronger than that of exocyclic amino group. Seemingly, both the -NH- of the azolium rings and the exocyclic N-NH_2_ are the potential candidates as the nucleophilic group attacking the carbon atom of the protonated DCA. Furthermore, together with the present experimental results, one of the reasons for the transformation reactions being thermodynamically favorable can be assigned to the covalent adducts formation. In consequence, the reaction could be further extended to other heterocyclic cations containing both dissociated protons and nucleophilic nitrogen atoms.

**Figure 6:**
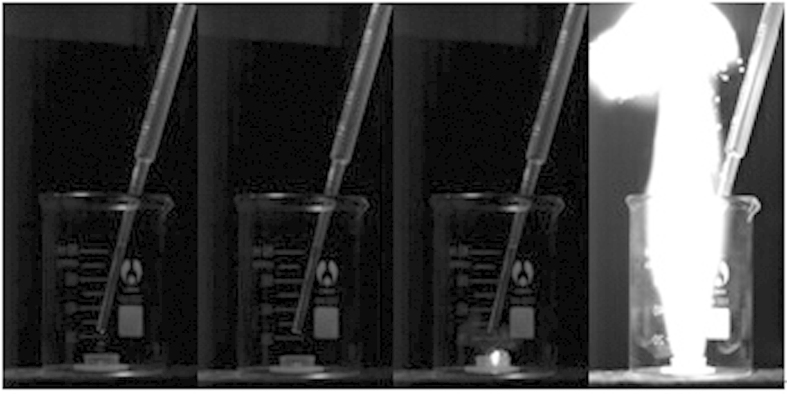
Hypergolic record of compound **8** by high-speed camera (a droplet of **8** into white fuming nitric acid).

Since DCA-ILs were shown to behave as hypergolic fuels^19^,^20^,^23^, droplet-ignition test is a common methodology to evaluate whether our cyanoguanidine-azoles possess hypergolic properties. Due to relatively-high melting points of most of the prepared cyanoguanidine-azoles (Table 5), only liquid compound **8** was selected for droplet test to assess its hypergolic property, where high-speed camera was used to record its hypergolic performance (Fig. 6). It was found that **8** spontaneously ignited upon its mixing with white fuming nitric acid (WFNA) or with red fuming nitric acid (RFNA), showing ignition delay (ID) time of 8 ms and 10 ms, with WFNA and RFNA, respectively. Thus, here we presented a novel type of hypergolic fuels. In future, on a platform of compound **8**, new cyanoguanidine-azole-based hypergolic liquids could be rationally designed and synthesized, by using azolium salts with relatively-low melting temperatures.

The present studies show that these cyanoguanidine-containing compounds all have high nitrogen content and high enthalpy of formation.

The cyanoguanidine group as the energetic functional moieties, could be further modified into even more energetic groups, such as azoles, and therefore are of high importance for future studies.

## Conclusion

A new family of compounds, containing both cyanoguanidine and azole moieties, was synthesized for the first time, by reacting azolium salts with silver dicyanamide. The resulted products were comprehensively characterized by multinuclear NMR, mass spectrometry, FTIR and X-ray crystallography. Also, DFT calculations were carried out to study the mechanism of this new transformation, showing essential factors influencing this reaction. The discovered new solid-state reaction could be extended to other heterocyclic ring systems possessing the reaction essentials. Additionally, liquid compound **8** was exhibited good hypergolic performance through droplet test, which stands for a novel type of hypergolic compound.

## Methods

### General methods

All materials were commercially available and used as received. IR spectra were recorded by using KBr pellets for solids on a Bruker tensor 27, spectrometer. ^1^H NMR and ^13^C NMR s spectroscopy were recorded on ARX-400 instrument with TMS as an internal standard. Elemental analyses were performed on an Elementar Vario EL(Germany). The crystal structure was determined by Rigaku RAXIS IP diffractometer and SHELXTL crystallographic software package of molecular structure. To determine the thermal stability of the described compound, a TA-DSC Q2000 differential scanning calorimeter was used.

### Silver dicyanamide

A solution of AgNO_3_ (3.40 g, 20 mmol) in distilled water (20 mL) was added dropwise to the solution of sodium dicyanamide (1.78 g, 20 mmol) in distilled water (40 mL) under stirring. After half an hour, the precipitate was filtered, and rinsed with 10 mL distilled water. The precipitate was dried naturally to obtain a white solid (silver dicyanamide). Yield: 3.2g, 92%;

### 1-(2-Cyanoamidino)-1,2,3-triazole (4)

A mixture of 1-H-1,2,3-triazolium hydrochloride (0.53 g, 5 mmol) and silver dicyanamide (1.04 g, 6 mmol) in water (50 mL) was stirred for 3 h at room temperature. After that time, formed AgCl precipitate was filtered out and washed with water (3 × 15 mL). Combined aqueous fractions were evaporated under vacuum, producing white solid residue. The resulted solid was kept at room temperature for additional 2 h and then purified by recrystallization from methanol, to yield pure **4** (0.55 g, 81%), as a white solid. MS m/z (ESI^+^): 136.9 [C_4_H_4_N_6_]^+^. Elemental analysis (%) calcd for C_4_H_4_N_6_: C, 35.29; H, 2.97; N, 61.76%; found C 34.92, H 3.21, N 61.36%. IR (KBr): 3470, 3309, 3207, 2202, 2159, 1653, 1601, 1485, 1396, 1259, 1231, 1167, 1135, 1062, 1013, 961, 844, 803, 745, 694, 636, 551, 462 cm^−1^. ^1^H NMR(acetone-d_6_) δ: 9.86, 9.69, 8.70, 7.99 ppm. ^13^C NMR (D_2_O): δ: 155.60, 130.96, 122.34, 63.44 ppm.

### 1-(2-Cyanoamidino)-1,2,4-triazole (5)

A mixture of 4-H-1,2,4-triazolium hydrochloride (0.844 g, 8 mmol) and silver dicyanamide (1.39 g, 8 mmol) in water (100 mL) was stirred for 4 h at room temperature. After filtration of formed AgCl precipitate, it was washed with water and combined aqueous fractions were evaporated under vacuum, producing white solid residue. The resulted solid was heated to 50 °C for 1 h and then purified by recrystallization from methanol to yield pure **5** (0.82 g, 75%), as a white solid. MS m/z (ESI^−^): 135.0 [C_4_H_4_N_6_]^−^. Elemental analysis (%) calcd. for C_4_H_4_N_6_: C, 35.30; H, 2.96; N, 61.74%; found C 35.16, H 2.85, N 61.70%. M.p. 57.4 °C. IR (KBr): 3335, 3221, 3130, 2198, 1674, 1607, 1509, 1484, 1431, 1387, 1332, 1279, 1189, 1121, 1097, 1003, 984, 961, 889, 802, 787, 693, 673, 634, 566 cm^−1^. ^1^H NMR(DMSO-d_6_) δ: 8.24, 6.55, 5.99 ppm. ^13^C NMR (DMSO-d_6_): δ: 167.8, 153.9, 144.9, 114.2 ppm.

### 1-(2-Cyanoamidino)-imidazole (6)

A mixture of imidazolium hydrochloride (0.732 g, 7 mmol) and silver dicyanamide (1.22 g, 7 mmol) in water (100 mL) was stirred for 4 h at room temperature. After filtration of formed AgCl precipitate, it was washed with water and combined aqueous fractions were evaporated under vacuum, producing brown solid residue. The resulted solid was heated to 50 °C for 2 h and then purified by recrystallization from methanol to yield pure **6** (0.71 g, 75%). MS m/z (ESI^−^): 133.9 [C_5_H_5_N_5_]^−^. Elemental analysis (%) calcd. for C_5_H_5_N_5_: C, 44.44; H, 3.73; N, 51.83%; found C 44.35, H 3.64, N 51.78%. M.p. 180.3 °C (decomp.). IR (KBr): 3219, 3152, 3129, 2202, 2182, 1607, 1479, 1444, 1349, 1306, 1221, 1137, 1087, 1010, 974, 911, 849, 809, 749, 720, 641, 566, 543, 502 cm^−1^. ^1^H NMR(DMSO-d_6_) δ: 8.43, 8.35, 7.75, 7.38, 7.09 ppm. ^13^C NMR (DMSO-d_6_): δ: 167.2, 154.9, 136.1, 130.4, 116.9 ppm.

### 1-(2-Cyanoamidino)-pyrazole (7)

A mixture of pyrazolium hydrochloride (0.837 g, 8 mmol) and silver dicyanamide (1.39 g, 8 mmol) in water (100 mL) was stirred for 4 h at room temperature. After filtration of formed AgCl precipitate, it was washed with water and combined aqueous fractions were evaporated under vacuum, producing white solid residue. The resulted solid was heated to 50 °C for 1.5 h and then purified by recrystallization from methanol to yield pure **7 (**0.86 g, 80%). MS m/z (ESI^−^): 134.0 [C_5_H_5_N_5_]^−^. Elemental analysis (%) calcd. for C_5_H_5_N_5_: C, 44.44; H, 3.73; N, 51.83%; found C 44.38, H 3.68, N 51.72%. M.p. 259.2 °C (decomp.). IR (KBr): 3311, 3140, 3099, 2260, 2200, 1683, 1600, 1535, 1497, 1410, 1383, 1213, 1158, 1078, 987, 941, 914, 766, 715, 643, 568, 538, 509 cm^−1^. ^1^H NMR (DMSO-d_6_) δ: 6.65, 5.87, 5.42, 4.09 ppm. ^13^C NMR (DMSO-d_6_): δ: 155.4, 144.4, 129.5, 114.6, 110.1 ppm.

### 3-Methyl-1-(3-cyanoguanidino)-1,2,3-triazole (8)

A mixture of 3-methyl-1-amino-1,2,3-triazolium iodide (1.13 g, 5 mmol) and silver dicyandiamide (1.04 g, 6 mmol) in water (60 mL) was stirred for 5 h at room temperature. After filtration of fomed AgI precipitate, it was washed with water (20 mL) and combined aqueous fractions were evaporated under vacuum, producing light yellow liquid. The resulted liquid could be solidified upon cooling and purified was conducted by crystallization from methanol at 4 ^o^C to yield pure **8** (0.69 g, 84%). MS m/z (ESI^+^): 166.1 [C_5_H_7_N_7_]^+^. Elemental analysis (%) calcd. for C_5_H_7_N_7_: C 36.36, H 4.28, N 59.38%; found C 35.98, H 4.42, N 58.91%. M.p. 8.6 °C. IR (KBr): 3346, 3192, 3141, 3110, 2168, 1647, 1525, 1462, 1401, 1384, 1326, 1273, 1221, 1101, 1066, 930, 817, 720, 656, 610, 530, 489, 429 cm^−1^; ^1^H NMR (MeOD) δ: 8.57, 8.44, 4.29 ppm. ^13^C NMR (MeOD): δ: 131.0, 127.5, 39.2 ppm.

### 4-(3-cyanoguanidino)-1,2,4-triazole (9)

A mixture of 4-NH_2_-1,2,4-triazolium hydrochloride (1.085 g, 9 mmol) and silver dicyanamide (1.565 g, 9 mmol) in water (100 mL) was stirred for 4 h at room temperature. After filtration of formed AgCl, it was washed with water and combined aqueous fractions were evaporated under vacuum, producing pink viscous liquid. The resulted liquid was heated to 102 °C for 2 h and purified by recrystallization from methanol to yield pure **9** (0.83 g, 61%), as a light yellow solid. MS m/z (ESI^−^): 150.5 [C_4_H_5_N_7_]^−^. Elemental analysis (%) calcd. for C_4_H_5_N_7_: C, 31.79; H, 3.33; N, 64.88%; found C 31.70, H 3.25, N 64.79%. M.p. 72.1 °C, decomp. 287.6 °C. IR (KBr): 3337, 3171, 2963, 2185, 1638, 1560, 1459, 1421, 1375, 1262, 1096, 1025, 865, 803, 744, 625 cm^−1^. ^1^H NMR(DMSO-d_6_) δ: 8.35, 6.99, 6.18 ppm. ^13^C NMR(DMSO-d_6_) δ:165.1, 161.9, 143.8, 139.9 ppm.

### 1-(3-cyanoguanidino)-1,2,3-triazole (10)

A mixture of 1-NH_2_-1,2,3-triazolium hydrochloride (0.965  g, 8 mmol) and silver dicyanamide (1.39 g, 8 mmol) in water (100 mL) was stirred for 4 h at room temperature. After filtration of formed AgCl, it was washed with water and combined aqueous fractions were evaporated under vacuum, producing yellow viscous liquid. The resuled liquid was heated to 102 °C for 1 h and purified by recrystallization from methanol to yield pure **10** (0.75 g, 62%), as a yellow solid. MS m/z (ESI^−^): 149.9 [C_4_H_5_N_7_]^−^. Elemental analysis (%) calcd. for C_4_H_5_N_7_: C, 31.79; H, 3.33; N, 64.88%; found C 31.68, H 3.22, N 64.80%. M.p. 214.3 °C (decomp.). IR (KBr): 3305, 3196, 2199, 2159, 1701, 1664, 1595, 1499, 1384, 1130, 1079, 972, 762, 744, 688, 636, 545 cm^−1^. ^1^H NMR (DMSO-d_6_) δ: 9.43, 7.10 ppm.

### 3,5-diamino-1-(2-cyanoamidino)-1,2,4-triazole (11)

A mixture of hydrazine dihydrochloride (0.525 g, 5 mmol) and silver dicyanamide (2.08 g, 12 mmol) in water (100 mL) was stirred for 4 h at room temperature. After filtration of formed AgCl, it was washed with water and combined aqueous fractions were evaporated under vacuum, producing orange solid residue. The resulted solid was kept at room temperature for additional 2 h and then purified by recrystallization from methanol to yield pure **8** (0.61 g, 73%), as an orange solid. MS m/z (ESI^+^): 166.9 [C_4_H_6_N_8_]^+^. Elemental analysis (%) calcd. for C_4_H_6_N_8_: C, 28.92; H, 3.65; N, 67.46%; found C 28.89, H 4.02, N 67.16%. M.p. 85.5 °C. IR (KBr): 3400, 3309, 3237, 3122, 2360, 2330, 2026, 1626, 1586, 1563, 1488, 1418, 1348, 1127, 1090, 967, 928, 808, 725, 670, 665, 651, 619, 527, 507 cm^−1^. ^1^H NMR (Acetone-d_6_) δ: 8.46, 7.63, 5.42 ppm; ^13^C NMR (D_2_O): δ: 157.3, 155.3, 130.9, 124.8, 122.3 ppm.

## Additional Information

**How to cite this article**: Liu, W. *et al.* Solid-state Reaction of Azolium Hydrohalogen Salts with Silver Dicyanamide–Unexpected Formation of Cyanoguanidine-azoles, Reaction Mechanism and Their Hypergolic Properties. *Sci. Rep.* **5**, 10915; doi: 10.1038/srep10915 (2015).

## Supplementary Material

Supplementary Information

## Figures and Tables

**Table 1:** Selected bond lengths /Å and bond angles /° of compound **4** and **8**.

**Bond lengths**	**4**	**8**
N(1)-C(1)	1.345(2)	1.368(4)
N(1)-N(2)	1.3270(16)	1.308(4)
N(2)-N(3)	1.3222(17)	1.366(3)
N(3)-C(2)	1.3528(19)	1.353(4)
C(1)-C(2)	1.364(2)	1.353(4)
N(4)-C(4)/ N(3)-C(3)	1.3321(18)	1.413(4)
N(5)-C(4)/ N(4)-C(3)	1.3490(17)	1.304(4)
N(7)-C(4)/ N(5)-C(3)	1.3448(18)	1.310(4)
N(5)-C(5)/ N(5)-C(4)	1.3124(18)	1.327(4)
N(6)-C(5)/ N(6)-C(4)	1.1624(18)	1.153(4)
Bond angles		
C(1)-N(1)-N(2)	112.80(12)	109.5(3)
N(3)-C(2)-C(1)	105.08(13)	104.6(3)
N(1)-C(1)-C(2)	105.70(13)	108.8(3)
N(3)-N(2)-N(1)	103.45(11)	106.0(2)
C(2)-N(3)-N(2)	112.96(12)	111.0(2)
N(5)-C(4)-N(7)/N(5)-C(3)-N(4)	122.88(12)	129.6(3)
C(5)-N(5)-C(4)/C(4)-N(5)-C(3)	116.79(11)	120.5(3)
N(4)-C(4)-N(7)/N(3)-C(3)-N(4)	113.96(12)	117.8(3)
N(4)-C(4)-N(5)/N(3)-C(3)-N(5)	123.17(12)	112.6(2)
N(6)-C(5)-N(5)/N(6)-C(4)-N(5)	175.06(15)	171.0(3)

**Table 2:** Selected bond lengths /Å and bond angles /° of compound **11**.

**Bond lengths**			
N(1)-C(1)	1.3863(16)	N(4)-C(1)	1.3212(17)
N(1)-N(2)	1.4085(15)	N(5)-C(2)	1.3733(18)
N(3)-C(1)	1.3279(17)	N(6)-C(3)	1.3135(18)
N(2)-C(2)	1.3220(18)	N(7)-C(4)	1.3203(17)
N(3)-C(2)	1.3608(17)	N(7)-C(3)	1.3244(17)
N(1)-C(3)	1.3803(17)	N(8)-C(4)	1.1585(18)
**Bond angles**			
C(1)-N(1)-N(2)	109.14(10)	N(4)-C(1)-N(3)	125.75(12)
C(2)-N(2)-N(1)	101.17(10)	N(4)-C(1)-N(1)	125.20(12)
C(1)-N(3)-C(2)	104.03(11)	N(2)-C(2)-N(5)	122.54(13)
N(3)-C(1)-N(1)	109.05(11)	N(3)-C(2)-N(5)	120.81(12)
N(2)-C(2)-N(3)	116.60(12)	N(6)-C(3)-N(7)	127.69(12)
C(3)-N(1)-C(1)	130.01(11)	N(6)-C(3)-N(1)	116.99(12)
C(3)-N(1)-N(2)	120.77(11)	N(7)-C(3)-N(1)	115.32(12)
C(4)-N(7)-C(3)	119.45(11)	N(8)-C(4)-N(7)	172.79(14)

**Table 3:** Crystal data and structure refinement details of **4**, **8** and **11**.

**Crystal**	**4**	**8**	**11·H_2_O**
Empirical formula	C_4_H_4_N_6_	C_5_H_7_N_7_	C_4_H_8_N_8_O_1_
Temperature (K)	173(2)K	153(2)K	153(2)K
Wavelength (Ǻ)	0.71073 A	0.71073 A	0.71073 A
Crystal system	Monoclinic	Monoclinic	Monoclinic
Space group	*P*2 (1) /*n*	*P*2 (1)*/c*	*P*2 (1) /*n*
Unit cell dimensions	*a* = 3.7428(17) Ǻ, *α*=90.00°	*a* = 9.378(4) Ǻ, *α* = 90.00°	*a* = 6.7142(15) Ǻ, *α* = 90.00°
	*b* = 19.927(8) Ǻ, *β* = 100.296(7)°	*b*=10.135(4)Ǻ, *β* = 113.553(4)°	*b* = 7.0332(16) Ǻ, *β* = 91.557(4)°
	*c* = 7.875(4) Ǻ, *γ* = 90.00°	*c* = 8.824(4) Ǻ, *γ* = 90.00°	*c* = 16.589(4) Ǻ, *γ* = 90.00°
Volume	577.9(4)	768.8(5) Ǻ^3^	783.1(3) Ǻ^3^
*Z*	4	4	4
Calculated density (g·cm^−3^)	1.565 g·cm^−3^	1.427 g·cm^−3^	1.562 g·cm^−3^
Absorption coefficient (mm^−1^)	0.115 mm^−1^	0.104 mm^−1^	0.123 mm^−1^
F(000)	280	344	384
Crystal size (mm)	0.44 × 0.19 × 0.15 mm	0.45 × 0.26 × 0.23 mm	0.28 × 0.20 × 0.08 mm
Theta range for data collection (°)	2.82° to 27.49°	3.11° to 27.99°	3.15° to 29.12°
Limiting indices	−4≤*h*≤4, −25≤*k*≤19, −10≤*l*≤10	−12≤*h*≤12, −11≤*k*≤13, −11≤*l*≤11	−8≤*h*≤9, −9≤*k*≤9, −17≤*l*≤22
Reflections collected / unique	4529/1314 [*R*(*int*) = 0.0543]	6014/1799 [*R*(*int*) = 0.0280]	6793/2100 [*R*(*int*) = 0.0309]
Completeness to theta = 25.03	99.2%	96.7%	99.3%
Reflections with *I*>2*σ* (*I*)	953	1424	1719
Goodness-of-fit on *F*2	1.002	1.001	1.001
Final R indices (*I*>2*sigma*(*I*))	*R1* = 0.0800, *wR2* = 0.1944	*R1* = 0.0400, *wR2* = 0.0959	*R1* = 0.0426, *wR2* = 0.1060
R indices (all data)	*R1* = 0.0981, *wR2* = 0.2059	*R1* = 0.0530, *wR2* = 0.1015	*R1* = 0.0567, *wR2* = 0.1151
CCDC	981619	981620	981618

**Table 4:** Reaction conditions of the transformation products.

**Compound**	**Temperature/°C**	**Time/h**	**Yield/%**
**4**	25	2	81
**5**	50	1	75
**6**	50	2	75
**7**	50	1.5	80
**8**	25	0.5	84
**9**	102	2	61
**10**	102	1	62
**11**	25	2	73

**Table 5:** Physicochemical properties of the transformation products.

**Comp.**	**T_m_ /°C**	**T_d_/°C**	**HOF/kJ·mol^−1^**	**ρ/g·cm^−3^**
**4**	—	195.3	318.7	1.56
**5**	57.4	>300	170	1.53
**6**	—	180.3	191.8	1.55
**7**	259.2	259.2	255.9	1.46
**8**	8.6	152.6	533.8	1.43
**9**	72.1	287.6	155	1.57
**10**	—	214.3	148.3	1.60
**11**	85.5	183.9	278.4	1.58
