# Supplementary material for: Solid-state Reaction of Azolium Hydrohalogen Salts with Silver Dicyanamide – Unexpected Formation of Cyanoguanidine-azoles, Reaction Mechanism and Their Hypergolic Properties
Source: Sci Rep. 2015 Jun 3;5:10915. doi: 10.1038/srep10915 (PMC4650654; doi:10.1038/srep10915)
Supplement: Supplementary Information [file srep10915-s1.pdf]

Supporting information:

Solid-state Reaction of Azolium Hydrohalogen Salts with Silver Dicyanamide – Unexpected Formation of Cyanoguanidine-azoles, Reaction Mechanism and Their Hypergolic Properties

Wei Liu, Qiu-han Lin, Yu-chuan Li, Peng-wan Chen, Tao Fang, Ru-bo Zhang* and Si-ping Pang*

Correspondence to zhangrubo@bit.edu.cn or pangsp@bit.edu.cn

Detailed computational information

All calculations were carried out using the Gaussian 09 program[1]. The heat of formation of the related compounds mentioned in this paper was computed by using an isodesmic reaction.[2] Based on the optimized structures, the enthalpy of the isodesmic reaction was obtained by combining the B3LYP/6-31 G * energy difference for the reaction and thermodynamic parameters, including the zero point energy (ZPE) and the thermal correction to the enthalpy (H_T).

The detonation performances of the related compounds were calculated by using EXPLO program (version 6.01)[3]. (Table S2).

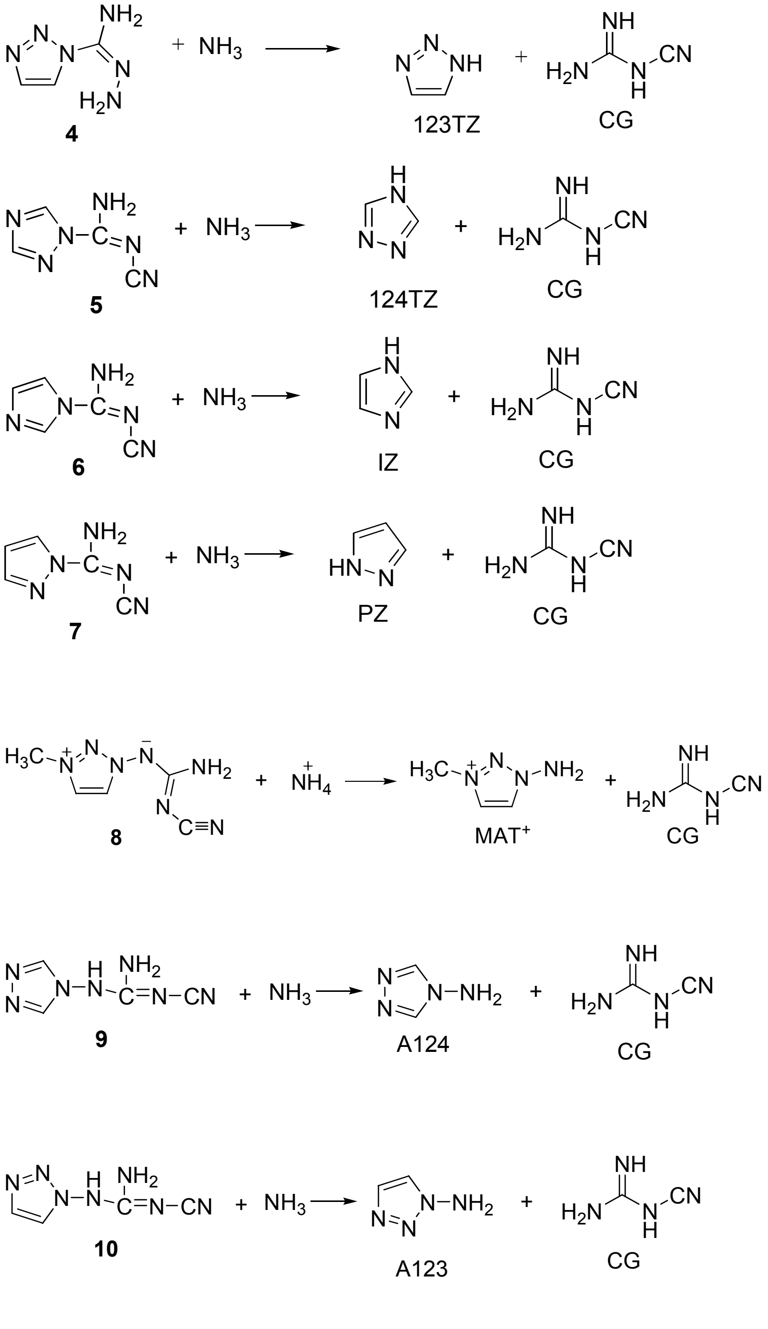

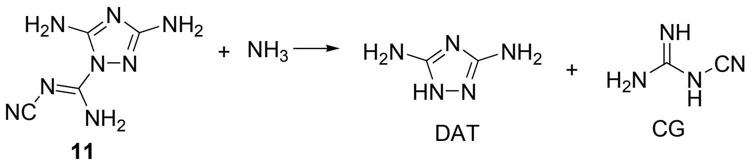

Scheme S1. Isodesmic reactions for calculating the heat of formation of transformation products.

Table S1. Calculated total energy (E_0), zero-point energy (ZPE), thermal correction (H_T), and heat of formation (HOF) of transformation products, and reference compounds.

Compd.	E_0/a.u.	ZPE/ a.u.	H_T/ a.u.	HOF/(kJ•mol^{-1})
NH_4^+	-56.529911	0.049867	0.003794	626.4[4]
CG	-295.834764	0.073775	0.007341	30[a]
NH_3	-56.18360515	0.034532	0.003803	-46.1[a]
**4**	-480.426713634	0.096648	0.009356	318.73
123TZ	-240.764795073	0.059101	0.00455	272[5]
**5**	-480.446608	0.097419	0.009237	170
124TZ	-240.789510	0.059101	0.004588	109[6]
**6**	-464.468220	0.109103	0.009427	191.8
IZ	-224.811991	0.071250	0.004723	129.5[a]
**7**	-464.440450	0.109001	0.009356	255.95
PZ	-224.790753	0.071403	0.004703	177.4[a]
**8**	-574.403385959	0.140807	0.011633	533.84
MAT^+	-335.166691496	0.117239	0.007853	951.57[5]
**9**	-535.439009	0.113375	0.010714	155.02
A124	-295.778548	0.076077	0.005861	105[6]
**10**	-535.418628	0.113763	0.010451	148.29
A123	-295.753157	0.075597	0.005925	110[5]
**11**	-590.529864236	0.131909	0.011804	278.36
DAT	-350.876613788	0.093602	0.00738	207.88[7]

[a] are refered to NIST data.

Table S2. The physicochemical properties of transformation products.

Compound	**4**	**5**	**6**	**7**	**8**	**9**	**10**	**11**
Formula	$C_4H_4N_6$	$C_4H_4N_6$	$C_5H_5N_5$	$C_5H_5N_5$	$C_5H_7N_7$	$C_4H_5N_7$	$C_4H_5N_7$	$C_4H_6N_8$
Molecuar Mass [g mol^{-1}]	136.11	136.11	135.13	135.13	165.17	151.13	151.13	166.16
IS [J] [a]	>40	>40	>40	>40	>40	>40	>40	>40
N [%][b]	66.6	61.7	51.8	51.8	59.4	64.9	64.9	67.4
T_m [°C] [c]	-	57.4	-	259.2	8.6	72.1	-	85.5
T_{dec} [°C] [d].	195.3	>300	180.3	259.2	152.6	287.6	214.3	183.9
OB[e]	-117.5	-117.5	-148.0	-148.0	-131	-111.2	-111.2	-105.9
Density [g cm^{-3}] [f]	1.565	1.532	1.551	1.459	1.427	1.566	1.601	1.581
$\Delta_f H°$[kJ mol-^{1}][g]	318.7	170	191.8	255.9	533.8	155	148.3	278.4
$\Delta_f U°$ [kJ kg^{-1}][h]	2341.5	1249.0	1419.4	1893.7	3231.8	1025.6	981.3	1675.5
$\Delta_E U°$ [kJ kg^{-1}] [i]	2824	1738	2012	2480	3885	1574	1530	2268
T_E [K] [j]	2143	1599	1641	1888	2510	1490	1462	1831
P [kbar] [k]	145	107	114	108	146	114	121	149
D [m s^{-1}] [l]	6720	5968	6140	5951	6793	6207	6342	6880
Gas vol. [L kg^{-1}] [m]	882	793	794	840	1033	825	822	928
Is [s] [n]	177	147	151	163	206	145	144	167

[a] Impact sensitivity. [b] Nitrogen Content. [c] Melting point. [d] Decomposition temperature. [e] CO_2 oxygen balance (OB) is an index of the deficiency or excess of oxygen in a compound required to convert all C into CO_2 and all H into H_2O. For a compound with the molecular formula of $C_aH_bN_cO_d$ (without crystal water), OB [%] = 1600[(d-2a-b/2)/MW], MW molecular weight of salt. [f] Measured Density [g·cm^{-3}]. Using ULTRAPYC 1200e v4.01, Automatic Density Analyzer [%]. [g] Molar enthalpy of the formation [kJ·mol^{-1}]. [h] Calculated energy of formation. [i] Energy of explosion. [j] Explosion temperature. [k] Detonation pressure. [l] Detonation velocity. [m] Assuming only gaseous products. [n] Specific impulse calculated at isobaric (60 bar) rocket conditions.

The present reaction paths were carried out with Gaussian 09 suites[1]. Structural optimizations and their energies were performed with a M06-2X/6-31+G(d,p) model with no limitation of symmetry. Vibrational frequencies and zero-point vibrational energies were computed at the same level. All the transition states found were verified

to be first-order saddle points by frequency calculations, and the vibrational mode with only one imaginary frequency corresponded to the reaction coordinate. Intrinsic reaction coordinate[8] calculations were used to determine which minima were connected by a particular TS. M06-2X functional[9] could give the accurate reaction energies and barriers for C, H, O and N-containing systems, which had been proven in our previous paper[10] through comparison with those calculated by the bench-mark CCSD(T) approach.

When there are the potentially dissociated protons are located on both the azolium ring and exocyclic N-NH_2 group such as 1-amino-1,2,3-triazolium, what transformation reaction path would occur? First, the reactive complex AD-0 was formed through release of heat of 6.4 kcal·mol^{-1}. Afterwards, the proton of azolium ring overrides a quite low barrier of 1.6 kcal mol^{-1} to the terminal nitrogen of DCA^{-}. The formed intermediate is quite unstable and it was resumed into the two separated constituents by solvation (AD-4). With respect to such a low barrier value, more possible image is postulated that both protonic solvent polarity and tunneling effects lead to the motion proton being transferred to DCA anion with no barrier. Our calculations show that the NH groups of the azolium ring in 1,2,3-triazolium and 1-amino-1,2,3- triazolium have the same deprotonated enthalpies, implying that their acidity is the same. Thus, the solvent-separated double molecules of the AD-4 could be encountered again to give the final product AD-P through the C-N bond formation and consistent proton transfer procedure. The barrier height is estimated by 25.1 kcal mol^{-1}. Another competitive reaction path involves the proton transfer of exocyclic N-NH_2 and synergistic C-N covalent bond formation to give the intermediate AD-2. The barrier of the small endothermic reaction is 26.9 kcal mol^{-1}, which is comparable to the barrier of the reaction AD-4 → AD-TS4 → AD-P. Starting from AD-2, the successive proton transfer reactions occur and also give the final product AD-P, seen in Figure S1.

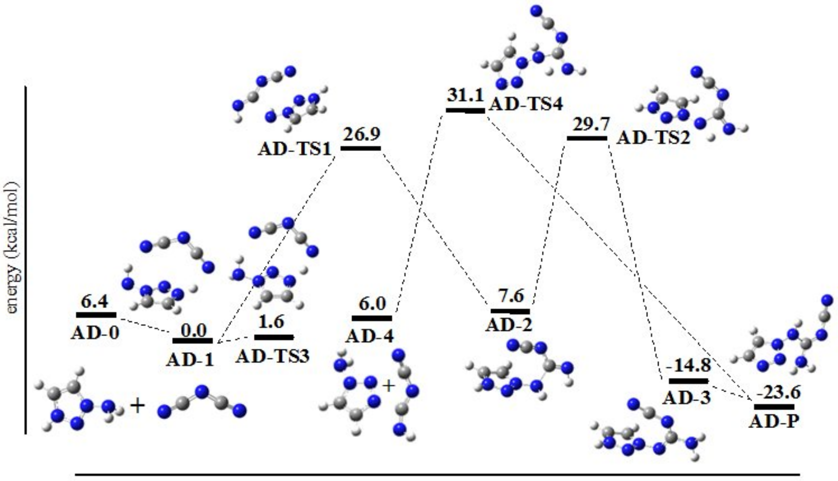

Figure S1. Theoretical calculation of reaction pathway for the transformation of l-amino-1,2,3-triazolium dicyanamide to compound **10**.

For the containing C-NH_2 3,5-diamino-1,2,4-triazolium cation, our calculations show that the reactive complexes HD-1 could be formed through $NH^{ring\cdots}N^{DCA}$ and $NH^{NH2\cdots}N^{DCA}$ hydrogen bonding, seen in Figure S2. The (N)H of the ring is partially

dissociated in HD-1. The phenomena show that its acidity is stronger than the (N)H of exocyclic C-NH_2 group. The dprotonated enthalpy of the (N)H of the ring is much less by ca. 7.0 kcal mol^{-1} than the (N)H of exocyclic C-NH_2 group. Thus, the (N)H of the ring is transferred to DCA anion favorably to the (N)H of exocyclic C-NH_2 group. The barrier is ca. 6.9 kcal mol^{-1} and the reaction energy is +6.4 kcal mol^{-1}. From HD-2, our calculations also show that the other NH group of the ring can added to DCA with a barrier height of ca. 33.9 kcal mol^{-1}. The addition of the NH group in C-NH_2 is excluded since the energy is consistently increases when the distance N atom of the NH group in C-NH_2 and C atom in DCA decreases. The following proton transfer reaction leads to the final product HD-P formation, whose barrier is estimated to be 32.9 kcal mol^{-1}. The net reaction energy from HD-1 to HD-P is -15.0 kcal mol^{-1}.

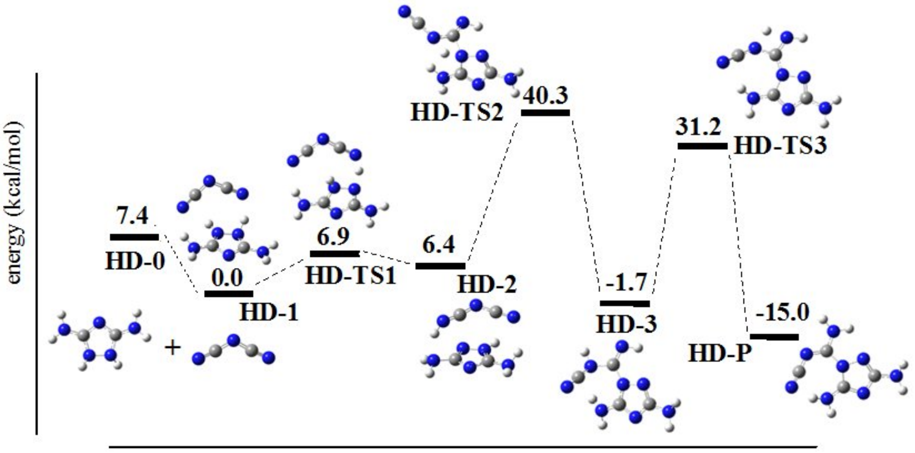

Figure S2. Theoretical calculation of reaction pathway for the transformation of 3,5-diamino -1,2,4-triazolium dicyanamide to compound **11**.

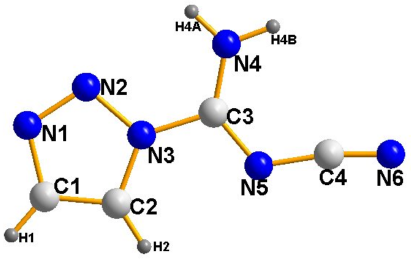

a)

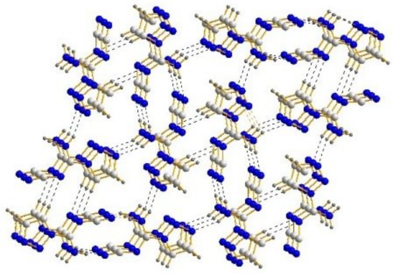

b)

Table S3. Energetic Summary:

	ZPE	E0(aug-cc-pVTZ)	Sum.	Relative value
	(a.u.)	(a.u.)	(a.u.)	(kcal mol^{-1})

MD-0	0.021499	-240.594106	-240.572607	
	0.11858	-337.293745	-337.175165	
			-577.747772	6.3
MD-1	0.141931	-577.8997881	-577.7578571	0
MD-TS1	0.139871	-577.8528033	-577.7129323	28.2
MD-2	0.144111	-577.8968219	-577.7527109	3.2
MD-TS2	0.138474	-577.8476854	-577.7092114	30.5
MD-P	0.143443	-577.9243812	-577.7809382	-14.5

TD-0	0.074129	-242.663567	-242.589438	
	0.021499	-240.594106	-240.572607	
			-483.162045	4.0
TD-1	0.094718	-483.2631529	-483.1684349	0
TD-TS1	0.090075	-483.1742845	-483.0842095	52.9
TD-2	0.096557	-483.2691454	-483.1725884	-2.6
TD-TS2	0.091391	-483.1416905	-483.0502995	74.1
TD-3	0.097677	-483.2761906	-483.1785136	-6.3
TD-TS3	0.092475	-483.2129177	-483.1204427	30.1
TD-P	0.097929	-483.2925456	-483.1946166	-16.4

HD-0	0.107418	-353.4382133	-353.3307953	
	0.021499	-240.594106	-240.572607	
			-593.9034023	7.4
HD-1	0.127688	-594.0428638	-593.9151758	0
HD-TS1	0.125194	-594.0283836	-593.9031896	7.5
HD-2	0.130405	-594.0345114	-593.9041064	6.9
HD-TS2	0.126083	-593.9761462	-593.8500632	40.9
HD-3	0.133092	-594.0526551	-593.9195631	-2.8
HD-TS3	0.127746	-593.9922136	-593.8644676	31.8
HD-P	0.133487	-594.0715959	-593.9381089	-14.4

AD-0	0.090477	-297.9861395	-297.8956625	
	0.021499	-240.594106	-240.572607	
			-538.4682695	6.4
AD-1	0.112663	-538.5910599	-538.4783969	0
AD-TS1	0.112151	-538.5477162	-538.4355652	26.9
AD-2	0.115398	-538.5816148	-538.4662168	7.6
AD-TS2	0.110836	-538.5419139	-538.4310779	29.7
AD-3	0.115889	-538.6178698	-538.5019808	-14.8
AD-TS3	0.108828	-538.5846917	-538.4758637	1.6
AD-4	0.032564	-241.0218988	-240.9893348	

	0.07715	-297.5566064	-297.4794564	
			-538.4687913	6
AD-TS4	0.110305	-538.5391682	-538.4288632	31.1
AD-P	0.115357	-538.6313304	-538.5159734	-23.6

2、Coordinates:

the protonated 1,2,3-triazolium

1\1\GINC-LOCALHOST\SP\RM062X\Aug-CC-pVTZ\C2H4N3(1+)\ROOT\18-Nov-2014\0 \\# m062x/aug-cc-pvtz scrf=(cpcm,solvent=water)\\Title Card Required\\ 1,1\C,0,-0.685211,0.948885,0.000158\N,0,-1.023104,-0.366447,0.000089\H ,0,-1.953506,-0.779923,0.000138\H,0,1.953424,-0.780114,0.000876\H,0,1. 410368,1.748631,-0.00026\H,0,-1.410275,1.74869,0.000309\N,0,-0.000059, -1.170371,-0.000746\C,0,0.685314,0.948814,-0.00018\N,0,1.023074,-0.366 535,0.000524\\Version=EM64L-G09RevA.01\State=1-A\HF=-242.6635673\RMSD= 4.122e-09\Dipole=0.0000019,0.6329448,0.0006991\Quadrupole=7.6739709,-1 .3159316,-6.3580393,-0.0003908,0.0011161,-0.0019187\PG=C01 [X(C2H4N3)]\\@

DCA anion

1\1\GINC-LOCALHOST\SP\RM062X\Aug-CC-pVTZ\C2N3(1-)\ROOT\15-Nov-2014\0\\ # m062x/aug-cc-pvtz scrf=(cpcm,solvent=water) geom=check guess=read\\T itle Card Required\\-1,1\C,0,-0.9529549393,-2.0052686451,-0.2806871798 \N,0,0.0874024544,-2.537110813,-0.3931887171\N,0,-2.1750254312,-1.5304 213743,-0.177676172\C,0,-2.3688645492,-0.2538098004,0.0708722947\N,0,- 2.6603845348,0.8621876328,0.2889957743\\Version=EM64L-G09RevA.01\State =1-A\HF=-240.5941059\RMSD=7.472e-09\Dipole=-0.3677786,-0.28782,-0.0519 749\Quadrupole=-1.693933,-5.4037433,7.0976763,8.8275844,1.7917735,-2.5 970636\PG=C01 [X(C2N3)]\\@

TD-1

1\1\GINC-LOCALHOST\SP\RM062X\Aug-CC-pVTZ\C4H4N6\ZHANGZH\17-Nov-2011\0\ \# m062x/aug-cc-pvtz scrf=(cpcm,solvent=water)\\Title Card Required\\0 ,1\C,0,2.69035,-0.654309,0.058089\C,0,2.616076,0.716501,-0.050988\N,0, 0.591102,-0.156284,0.057553\H,0,3.554965,-1.297504,0.091883\H,0,3.3607 59,1.491406,-0.131863\N,0,1.424493,-1.147107,0.120678\N,0,1.290733,0.9 6728,-0.044934\C,0,-1.900596,1.224711,0.028935\N,0,-1.437497,2.270571, -0.177671\N,0,-2.458071,0.05994,0.34774\C,0,-1.94419,-1.025641,0.01884 \N,0,-1.644855,-2.134011,-0.328588\H,0,-0.819261,-2.624167,0.004572\H, 0,0.772355,1.839977,-0.117294\\Version=EM64L-G09RevA.01\State=1-A\HF=- 483.2631529\RMSD=5.531e-09\Dipole=1.6990111,-0.353212,0.1323125\Quadru pole=5.2014291,-2.6332076,-2.5682215,6.4197939,-0.7490001,-0.7625947\P G=C01 [X(C4H4N6)]\\@

TD-TS1

1\1\GINC-LOCALHOST\SP\RM062X\Aug-CC-pVTZ\C4H4N6\ZHANGZH\17-Nov-2011\0\ \#

m062x/aug-cc-pvtz scrf=(cpcm,solvent=water)\\Title Card Required\\0
,1\C,0,2.329728,-0.356763,0.596633\C,0,1.089116,-0.272686,1.115741\N,0
,1.027614,-0.186078,-1.145889\H,0,3.289164,-0.460108,1.077932\H,0,0.71
1514,-0.27184,2.127257\N,0,2.228485,-0.301027,-0.798352\N,0,0.223867,-
0.184772,0.018075\C,0,-2.153432,-0.718105,-0.007209\N,0,-1.827086,-1.8
6346,-0.045806\N,0,-2.126482,0.555746,0.041119\C,0,-0.857271,1.024906,
0.015192\N,0,-0.441655,2.203395,0.008963\H,0,0.569314,2.301209,-0.0239
17\H,0,-0.612035,-1.200003,-0.050189\\Version=EM64L-G09RevA.01\State=1
-A\HF=-483.1742845\RMSD=4.033e-09\Dipole=1.8018229,-0.3310007,1.743846
3\Quadrupole=2.0461118,-5.7464489,3.7003371,1.5630444,5.2453981,-1.198
7325\PG=C01 [X(C4H4N6)]\\@

TD-2

1\1\GINC-LOCALHOST\SP\RM062X\Aug-CC-pVTZ\C4H4N6\ZHANGZH\17-Nov-2011\0\ \#
m062x/aug-cc-pvtz scrf=(cpcm,solvent=water)\\Title Card Required\\0
,1\C,0,2.491859,-0.661337,0.238881\C,0,1.835856,0.519121,0.467453\N,0,
0.492186,-0.943782,-0.531063\H,0,3.503696,-0.945496,0.479953\H,0,2.128
907,1.438427,0.948593\N,0,1.62998,-1.524239,-0.373592\N,0,0.587821,0.3
01825,-0.025112\C,0,-2.083395,-0.515229,0.087308\N,0,-2.573007,-1.5560
69,0.460287\N,0,-1.766637,0.640182,-0.19741\C,0,-0.519565,1.223633,-0.
101897\N,0,-0.381208,2.478151,-0.10584\H,0,0.599074,2.753029,-0.100292
\H,0,-2.504153,-2.415568,-0.069615\\Version=EM64L-G09RevA.01\State=1-A
\HF=-483.2691454\RMSD=9.505e-09\Dipole=1.3325825,0.0711603,0.5216612\Q
uadrupole=3.9601525,-2.7869004,-1.1732521,10.6595822,4.3684587,1.30477
54\PG=C01 [X(C4H4N6)]\\@

TD-TS2

1\1\GINC-LOCALHOST\SP\RM062X\Aug-CC-pVTZ\C4H4N6\ZHANGZH\17-Nov-2011\0\ \#
m062x/aug-cc-pvtz scrf=(cpcm,solvent=water)\\Title Card Required\\0
,1\C,0,2.572331,-0.526037,0.13583\C,0,1.891102,0.658069,0.243083\N,0,0
.510976,-1.006232,-0.281628\H,0,3.622448,-0.731785,0.267039\H,0,2.1651
07,1.675813,0.467912\N,0,1.686557,-1.517197,-0.184296\N,0,0.606287,0.3
09341,-0.021579\C,0,-1.941622,-0.777579,-0.049013\N,0,-2.632052,-1.644
275,0.387535\N,0,-1.743688,0.501515,-0.355871\C,0,-0.54381,1.147636,-0
.078942\N,0,-0.370231,2.390935,0.062861\H,0,-1.260661,2.881282,-0.0008
04\H,0,-2.799838,-0.076456,0.510948\\Version=EM64L-G09RevA.01\State=1-
A\HF=-483.1416905\RMSD=4.636e-09\Dipole=-0.0014208,1.6621173,0.5312718
\Quadrupole=8.0149173,-7.6697759,-0.3451414,-3.2583508,0.1936701,0.707
1339\PG=C01 [X(C4H4N6)]\\@

TD-3

1|1|UNPC-ROOT-PC|SP|RM062X|Aug-CC-pVTZ|C4H4N6|ROOT|24-Nov-2014|0||#
m062x/aug-cc-pvtz scrf=(cpcm,solvent=water) geom=check guess=read||Title
Card Required||0,1|C,0,2.5312358353,-0.4557974873,0.3039929626|C,0,1.

6959106725,0.5665985622,0.659532851|N,0,0.7134236213,-0.7819637561,-0.
8296602592|H,0,3.5295343476,-0.6808752171,0.6430808323|H,0,1.779613444
4,1.3877440866,1.3539058817|N,0,1.8876815206,-1.2472461249,-0.60865987
71|N,0,0.5669772945,0.3261809289,-0.0639540594|C,0,-1.9481223424,-0.85
63328781,0.2231694522|N,0,-2.135761715,-1.921514672,0.6377127151|N,0,-
1.7758223812,0.3875866055,-0.260072462|C,0,-0.601992071,1.1243069782,-
0.1521088289|N,0,-0.6429502509,2.3860566283,-0.1562362344|H,0,0.286613
7165,2.7970757309,-0.2026276713|H,0,-2.6021178721,0.9493795449,-0.4292
986024||Version=IA32W-G09RevA.02|State=1-A|HF=-483.2759833|RMSD=5.984e
-009|Dipole=0.7676042,2.6700118,0.6463985|Quadrupole=7.3849363,-7.4749
783,0.0900419,-0.8145905,6.6264954,0.6821455|PG=C01 [X(C4H4N6)]||@

TD-TS3

1|1|UNPC-ROOT-PC|SP|RM062X|Aug-CC-pVTZ|C4H4N6|ROOT|24-Nov-2014|0||#
m062x/aug-cc-pvtz scrf=(cpcm,solvent=water) geom=check guess=read||Title
Card Required||0,1|C,0,2.6338439763,-0.3439547286,0.3403092771|C,0,1.
7788084125,0.6881088378,0.5855098213|N,0,0.7431713421,-0.9653334397,-0
.5264456431|H,0,3.6722517411,-0.4602710684,0.6053386569|H,0,1.87717739
28,1.6271866609,1.1062933055|N,0,1.9533101935,-1.3244952733,-0.3434392
308|N,0,0.5991008874,0.2688858825,0.0330171497|C,0,-2.2117521036,-0.86
03653502,0.0016970796|N,0,-2.6460333085,-1.9132184821,0.225850233|N,0,
-1.8101092123,0.3836354451,-0.2823723044|C,0,-0.6122958947,0.936425475
4,-0.0379711474|N,0,-0.8357053871,2.2128758564,0.1172821765|H,0,-0.124
9156921,2.9218844259,-0.0190029065|H,0,-2.0573813874,1.6852922183,-0.1
253672674||Version=IA32W-G09RevA.02|State=1-A|HF=-483.2129177|RMSD=7.1
64e-009|Dipole=2.1355555,3.7421354,0.6634523|Quadrupole=0.1636195,-1.4
032591,1.2396396,-3.4407695,4.1919335,-0.2996184|PG=C01 [X(C4H4N6)]||@

TD-P

1|1|UNPC-ROOT-PC|SP|RM062X|Aug-CC-pVTZ|C4H4N6|ROOT|24-Nov-2014|0||#
m062x/aug-cc-pvtz scrf=(cpcm,solvent=water) geom=check guess=read||Title
Card Required||0,1|C,0,2.5227299747,-0.3655901643,0.2415602038|C,0,1.
6636752262,0.628719459,0.616652323|N,0,0.6565413319,-0.8163788404,-0.7
662307547|H,0,3.5496200037,-0.5335534653,0.5239182807|H,0,1.7440840413
,1.4745760905,1.2807683488|N,0,1.8635010963,-1.2155697732,-0.606735647
3|N,0,0.5033354652,0.3095893745,-0.0291300211|C,0,-2.0371541411,-0.854
520164,0.2596363906|N,0,-2.2954802861,-1.9607417079,0.4992686805|N,0,-
1.8854120629,0.4529069181,0.0135702992|C,0,-0.7307195943,1.0027288684,
-0.0367438804|N,0,-0.5913209307,2.3510466209,-0.0552347637|H,0,0.20734
23206,2.7507068268,-0.5258646545|H,0,-1.4549562349,2.8753248669,-0.084
5047447||Version=IA32W-G09RevA.02|State=1-A|HF=-483.2925456|RMSD=8.516
e-009|Dipole=1.9062471,4.4753226,0.4534468|Quadrupole=-1.7304113,1.638
4421,0.0919693,-5.1639362,5.0203586,-1.3283891|PG=C01 [X(C4H4N6)]||@

1-amino-3-methyl-1,2,3-triazolium cation

1\1\GINC-LOCALHOST\SP\RM062X\Aug-CC-pVTZ\C3H7N4(1+)\ROOT\15-Nov-2014\0 \\# m062x/aug-cc-pvtz scrf=(cpcm,solvent=water) geom=check guess=read\ \Title Card Required\\ 1,1\C,0,0.2774865308,1.1474767925, 1.186076975\C,0,1.0726841822,0.0436611823,1.3521999499\N,0,1.1598838613,0.6507462544 ,-0.7851300552\H,0,-0.329460829,1.7124281558,1.8773490368 \H,0,1.320743 1101,-0.573687023,2.2021662361\N,0,0.3760744779,1.4621114023,-0.132897 8013\N,0,1.5765174882,-0.2029916167,0.1183873367\ N,0,2.4395287476,-1.2459076461,-0.1784247558\H,0,3.3131964722,-0.8660637328,-0.5359856 978\C ,0,-0.2670225923,2.579078677,-0.8406639309\H,0,-0.1017569239,2.4272442 661,-1.9054047759\H,0,-1.3321781586,2.5653997918,-0.6125438759\H,0,0.1 887706887,3.5131980311,-0.5120168104\H,0,2.0055959448,-1.8533825347,-0 .8698618311\\Version=EM64L-G09RevA.01\State=1-A\HF=-337.2937445\RMSD=6 .530e-09\Dipole=-0.3647587, 0.3901641,0.2928354\Quadrupole=-0.816374,2. 3752452,-1.5588712,-6.7658086,-1.7626932,0.6717211\PG=C01 [X(C3H7N4)]\ \@

MD-1

1\1\GINC-LOCALHOST\SP\RM062X\Aug-CC-pVTZ\C5H7N7\ROOT\14-Nov-2014\0\\# m062x/aug-cc-pvtz scrf=(cpcm,solvent=water)\\Title Card Required\\0,1\ C,0,0.249128,1.111366,1.159028\C,0,1.091668,0.043318,1.334753\N,0,1.29 8246,0.746699,-0.756991\H,0,-0.487066,1.572454,1.798133\H,0,1.287315,- 0.619483,2.161131\N,0,0.435993,1.503019,-0.122734\N,0,1.693396,-0.1187 04,0.134861\N,0,2.556529,-1.157415,-0.17097\H,0,3.065854,-0.884051,-1. 007457\C,0,-0.237193,2.598013,-0.822965\H,0,-0.025082,2.479972,-1.8837 1\H,0,-1.306449,2.49101,-0.626324\H,0,0.152144,3.548528,-0.456895\C,0, -0.986261,-2.00013,-0.281633\N,0,0.089523,-2.460936,-0.377155\N,0,-2.2 13433,-1.564526,-0.181964\C,0,-2.379465,-0.286437,0.062176\N,0,-2.5801 91,0.847606,0.286892\H,0,1.92558,-1.955414,-0.37661\\Version=EM64L-G09 RevA.01\State=1-A\HF=-577.8997881\RMSD=3.485e-09\Dipole=3.165483,2.762 8067,0.4857008\Quadrupole=-7.9324311,0.5366309,7.3958002,-0.6564621,-0 .5904246,-1.9819873\PG=C01 [X(C5H7N7)]\\@

MD-TS1

1\1\GINC-LOCALHOST\SP\RM062X\Aug-CC-pVTZ\C5H7N7\ROOT\14-Nov-2014\0\\# m062x/aug-cc-pvtz scrf=(cpcm,solvent=water)\\Title Card Required\\0,1\ C,0,1.70067,-0.67781,1.117006\C,0,0.460955,-1.260417,1.12118\N,0,0.896 627,-0.579356,-0.949117\H,0,2.408774,-0.494847,1.90904\H,0,-0.143527,- 1.71244,1.888057\N,0,1.925162,-0.298303,-0.1542\N,0,0.011534,-1.155158 ,-0.167237\N,0,-1.221362,-1.489247,-0.554341\H,0,-1.208022,-1.401738,- 1.572568\C,0,3.006702,0.52173,-0.677815\H,0,3.249953,0.172779,-1.68088 3\H,0,2.677225,1.562521,-0.694742\H,0,3.86904,0.407387,-0.021806\C,0,- 2.364588,0.335246,-0.109924\N,0,-3.459726,-0.115235,0.209522\N,0,-1.60 9805,1.297115,-0.426129\C,0,-0.521345,1.72077,0.184486\N,0,0.457033,2. 162206,0.642736\H,0,-3.544054,-1.124935,0.154656\\Version=EM64L-G09Rev

A.01\State=1-A\HF=-577.8528033\RMSD=4.180e-09\Dipole=2.4327045,-1.7772
204,0.3445549\Quadrupole=6.2371671,-8.7335719,2.4964048,1.9505454,1.18
06305,-4.3104332\PG=C01 [X(C5H7N7)]\\@

MD-2

1\1\GINC-LOCALHOST\SP\RM062X\Aug-CC-pVTZ\C5H7N7\ROOT\24-Nov-2014\0\\#
m062x/aug-cc-pvtz scrf=(cpcm,solvent=water) geom=check guess=read\\Tit
le Card Required\\0,1\C,0,1.8976403256,-0.6815800723,1.1113899264\C,0,
0.6248752937,-1.0711936557,1.4204888461\N,0,0.6785528142,-0.521564079,
-0.7298699725\H,0,2.7841692836,-0.5581557542,1.7112720118\H,0,0.137381
2564,-1.3616711288,2.3361265931\N,0,1.8574940105,-0.3581193335,-0.2057
704567\N,0,-0.0662244392,-0.9435078883,0.262572043\N,0,-1.4389876401,-
1.030931261,0.1656496213\H,0,-1.7205159552,-1.8255543506,-0.4048843153
\C,0,2.8720581325,0.3677042485,-0.9668578901\H,0,2.7404795506,0.128618
8703,-2.020345849\H,0,2.6990454331,1.4310141912,-0.7799572875\H,0,3.85
57640567,0.0561972427,-0.6191178934\C,0,-2.1359359577,0.1693454953,-0.
3396607669\N,0,-3.2065901285,-0.1769299852,-0.9493694926\N,0,-1.674944
4221,1.4021427859,-0.0765171361\C,0,-0.5087637279,1.6990561259,0.42216
77144\N,0,0.5423925372,2.0433192958,0.8267368803\H,0,-3.7223817333,0.6
618300929,-1.2065599465\\Version=EM64L-G09RevA.01\State=1-A\HF=-577.89
68219\RMSD=5.438e-09\Dipole=4.2000098,-2.9644076,0.4443891\Quadrupole=
3.4893866,-6.2192105,2.7298239,-3.3859588,-3.4125738,-6.5894085\PG=C01
[X(C5H7N7)]\\@

MD-TS2

1\1\GINC-LOCALHOST\SP\RM062X\Aug-CC-pVTZ\C5H7N7\ROOT\24-Nov-2014\0\\#
m062x/aug-cc-pvtz scrf=(cpcm,solvent=water) geom=check guess=read\\Tit
le Card Required\\0,1\C,0,1.9125759652,-0.7768118533,1.1393894525\C,0,
0.6381816765,-1.0745602225,1.5387874072\N,0,0.5672514753,-0.4708636361
,-0.5923156742\H,0,2.8509500942,-0.759606555,1.6692767122\H,0,0.201912
3975,-1.3570390481,2.4817565691\N,0,1.8015363253,-0.4096198911,-0.1596
523713\N,0,-0.1331792467,-0.8526705113,0.4443090925\N,0,-1.5019943238,
-0.8546284822,0.4511285881\H,0,-2.3812486693,-1.4402869475,-0.26452479
89\C,0,2.8048886908,0.2322018668,-1.002120371\H,0,2.6477152689,-0.0904
103881,-2.0299970904\H,0,2.6721293969,1.312772769,-0.9117037429\H,0,3.
7912503266,-0.0706532772,-0.6539876586\C,0,-2.1108538568,0.2211343618,
-0.2817498138\N,0,-3.0804328349,-0.4419480472,-0.8746790972\N,0,-1.781
5891822,1.4975515879,-0.3051046665\C,0,-0.6785388282,1.8668451741,0.31
93615676\N,0,0.3268558774,2.2207321379,0.8057782941\H,0,-3.8644056026,
0.0562935722,-1.2785958185\\Version=EM64L-G09RevA.01\State=1-A\HF=-577
.8476854\RMSD=6.209e-09\Dipole=3.3127703,-2.6921807,0.2253792\Quadrupo
le=10.6098961,-11.3339321,0.724036,-3.1472882,-0.8193666,-5.5917651\PG
=C01 [X(C5H7N7)]\\@

MD-P

1\1\GINC-LOCALHOST\SP\RM062X\Aug-CC-pVTZ\C5H7N7\ROOT\24-Nov-2014\0\\#
m062x/aug-cc-pvtz scrf=(cpcm,solvent=water) geom=check guess=read\\Tit
le Card Required\\0,1\C,0,1.8414817791,-0.659773566,1.1763013085\C,0,0
.5475877298,-1.0237037085,1.4370074962\N,0,0.7004441167,-0.5351818165,
-0.7175986978\H,0,2.7058922741,-0.5539875357,1.8110592215\H,0,0.021837
4589,-1.3084616584,2.3320908451\N,0,1.8695743697,-0.3745145001,-0.1455
266788\N,0,-0.1009571733,-0.8980957345,0.2504248085\N,0,-1.4304403038,
-1.1165422893,0.0465280467\C,0,2.9256381424,0.295718003,-0.8939182734\
H,0,2.872968434,-0.0371287059,-1.929220014\H,0,2.7434130823,1.37120609
07,-0.8220313223\H,0,3.8856078251,0.0265755031,-0.4550923773\C,0,-2.10
63977405,0.0250808548,-0.2146250278\N,0,-3.4073746447,-0.1989414792,-0
.5294898744\N,0,-1.7468010668,1.3060371118,-0.192363506\C,0,-0.5556724
639,1.7359281911,0.1565742988\N,0,0.4812518847,2.204147793,0.440420619
\H,0,-4.0040411881,0.5921647106,-0.6971330538\H,0,-3.7434466656,-1.141
6401141,-0.6208942088\\Version=EM64L-G09RevA.01\State=1-A\HF=-577.9243
812\RMSD=7.106e-09\Dipole=1.8907076,-2.2588787,0.4556454\Quadrupole=16
.91362,-15.9251838,-0.9884362,-2.1471534,1.6412772,-4.245498\PG=C01 [X
(C5H7N7)]\\@

3,5-diamino-1,2,4-triazolium

1\1\GINC-LOCALHOST\SP\RM062X\Aug-CC-pVTZ\C2H6N5(1+)\ROOT\15-Nov-2014\0
\\# m062x/aug-cc-pvtz scrf=(cpcm,solvent=water) geom=check guess=read\
\Title Card Required\\1,1\N,0,0.4866663421,-0.7634058314,0.0779500205\
N,0,0.3165720328,0.6148902865,-0.1044352892\H,0,1.1171780778,3.1288282
866,-0.1587227719\C,0,1.5851360603,1.1346518165,-0.0354215198\N,0,1.83
3247555,2.4340882541,-0.0060260039\N,0,2.5236989093,0.1901135333,0.005
5014253\C,0,1.8450278696,-0.9559377433,0.0321949972\N,0,2.4047901073,-
2.1547210896,0.0099654673\H,0,2.7974716096,2.7340879876,0.04906614\H,0
,-0.4418418448,1.0270155442,0.4321077361\H,0,1.8783928752,-3.004244928
6,0.1512439141\H,0,-0.1375786881,-1.3480682293,-0.4710733231\H,0,3.413
925094,-2.2090728867,-0.0281577927\\Version=EM64L-G09RevA.01\State=1-A
\HF=-353.4382133\RMSD=4.262e-09\Dipole=-1.124799,-0.140448,-0.0107226\
Quadrupole=-2.2490231,14.4524547,-12.2034316,-2.1210163,-0.0076165,0.7
440795\PG=C01 [X(C2H6N5)]\\@

HD-1

1\1\GINC-LOCALHOST\SP\RM062X\Aug-CC-pVTZ\C4H6N8\ZHANGZH\14-Nov-2011\0\ \#
m062x/aug-cc-pvtz scrf=(cpcm,solvent=water)\\Title Card Required\\0
,1\N,0,0.551509,-0.750848,0.018401\N,0,0.387433,0.609575,-0.049516\H,0
,1.090225,3.107083,0.034577\C,0,1.615145,1.134999,-0.037682\N,0,1.8508
32,2.458776,-0.105502\N,0,2.562776,0.189419,0.00754\C,0,1.870501,-0.95
7668,0.036201\N,0,2.433519,-2.180518,0.117635\H,0,2.788665,2.768388,0.
093423\H,0,-0.544397,1.074747,0.000191\H,0,1.875257,-2.999848,-0.06590

2\H,0,-0.281713,-1.393234,-0.028318\H,0,3.422934,-2.232646,-0.066855\C
,0,-2.603523,-1.195085,-0.04977\N,0,-1.84424,-2.089848,-0.108366\N,0,-
3.417483,-0.172504,0.017039\C,0,-2.7528,0.956642,0.060608\N,0,-2.11390
6,1.941975,0.099447\\Version=EM64L-G09RevA.01\State=1-A\HF=-594.042863
8\RMSD=5.895e-09\Dipole=3.8337821,0.0282919,-0.0271546\Quadrupole=-6.5
461364,7.7987045,-1.2525681,-1.6251347,-0.2799592,1.7204219\PG=C01 [X(
C4H6N8)]\\@

HD-TS1

1\1\GINC-LOCALHOST\SP\RM062X\Aug-CC-pVTZ\C4H6N8\ZHANGZH\14-Nov-2011\0\ \#
m062x/aug-cc-pvtz scrf=(cpcm,solvent=water)\\Title Card Required\\0
,1\N,0,-1.131563,-0.715366,0.804028\N,0,-0.218339,0.332955,0.857319\H,
0,0.981237,2.492084,0.011272\C,0,-0.680945,1.354343,0.107351\N,0,-0.03
766,2.533847,-0.0034\N,0,-1.843609,1.037262,-0.460883\C,0,-2.066658,-0
.218956,-0.024272\N,0,-3.114594,-0.956191,-0.444824\H,0,-0.438975,3.15
9153,-0.687444\H,0,0.446467,0.392893,1.615528\H,0,-3.363419,-1.793197,
0.056318\H,0,-0.398063,-1.76443,0.342431\H,0,-3.823001,-0.488842,-0.98
6774\C,0,1.569516,-1.732655,-0.165551\N,0,0.534492,-2.309643,-0.208027
\N,0,2.706804,-1.159472,-0.089694\C,0,2.71245,0.160093,-0.21458\N,0,2.
731551,1.325949,-0.250094\\Version=EM64L-G09RevA.01\State=1-A\HF=-594.
0283836\RMSD=5.405e-09\Dipole=-2.3969029,0.1120041,0.5611618\Quadrupol
e=0.0110501,-0.9028177,0.8917677,3.2040192,2.6105721,-1.3551479\PG=C01
[X(C4H6N8)]\\@

HD-2

1\1\GINC-LOCALHOST\SP\RM062X\Aug-CC-pVTZ\C4H6N8\ZHANGZH\14-Nov-2011\0\ \#
m062x/aug-cc-pvtz scrf=(cpcm,solvent=water)\\Title Card Required\\0
,1\N,0,1.023156,-0.387109,1.317263\N,0,-0.132327,-1.082905,1.083558\H,
0,-2.109216,-1.915841,-0.483015\C,0,-0.160731,-1.527545,-0.18706\N,0,-
1.185001,-2.275643,-0.706247\N,0,0.952601,-1.168833,-0.813614\C,0,1.62
4573,-0.492447,0.153916\N,0,2.806835,0.199494,-0.133147\H,0,-1.066818,
-2.424905,-1.699852\H,0,-0.825524,-1.188919,1.808413\H,0,3.388846,0.31
555,0.689322\H,0,1.784102,1.819989,-0.601158\H,0,3.319095,-0.242897,-0
.888075\C,0,-0.152558,2.018968,-0.210596\N,0,0.880205,2.301437,-0.7426
75\N,0,-1.266634,1.922688,0.343798\C,0,-2.164886,0.980774,0.0867\N,0,-
2.988674,0.17066,-0.046563\\Version=EM64L-G09RevA.01\State=1-A\HF=-594
.0345114\RMSD=7.873e-09\Dipole=1.5014805,-0.251674,-0.0127081\Quadrupo
le=2.7250665,-2.4239802,-0.3010862,3.450006,-0.5695261,0.1816182\PG=C0
1 [X(C4H6N8)]\\@

HD-TS2

1\1\GINC-LOCALHOST\SP\RM062X\Aug-CC-pVTZ\C4H6N8\ZHANGZH\14-Nov-2011\0\ \#
m062x/aug-cc-pvtz scrf=(cpcm,solvent=water)\\Title Card Required\\0
,1\N,0,1.285239,-1.053793,-0.516071\N,0,0.233105,-0.127897,-0.390097\H

,0,-0.941836,2.093102,0.224983\C,0,0.833551,1.1006,0.073473\N,0,0.0634
71,2.160623,0.318541\N,0,2.11869,0.987773,0.167007\C,0,2.337437,-0.339
003,-0.198687\N,0,3.592321,-0.822404,-0.190654\H,0,0.490913,3.005638,0
.663371\H,0,-0.709398,0.065467,-1.198187\H,0,3.768046,-1.735062,-0.578
678\H,0,-0.149399,-1.4853,1.79674\H,0,4.354809,-0.175084,-0.079941\C,0
,-1.012154,-0.568289,0.386768\N,0,-1.071391,-1.223438,1.45079\N,0,-1.8
98532,-0.023684,-0.496011\C,0,-3.22325,-0.142731,-0.388696\N,0,-4.3838
51,-0.193212,-0.352852\\Version=EM64L-G09RevA.01\State=1-A\HF=-593.976
1462\RMSD=4.451e-09\Dipole=4.4245219,1.7173873,-0.2377018\Quadrupole=-
12.9347636,10.2419387,2.6928249,-8.3976076,-0.7805093,2.2923199\PG=C01
[X(C4H6N8)]\\@

HD-3

1\1\GINC-LOCALHOST\SP\RM062X\Aug-CC-pVTZ\C4H6N8\ZHANGZH\15-Nov-2011\0\ \#
m062x/aug-cc-pvtz scrf=(cpcm,solvent=water)\\Title Card Required\\0
,1\N,0,1.359894,-1.091689,-0.032715\N,0,0.327387,-0.181474,0.054982\H,
0,-0.602879,2.249588,0.882484\C,0,0.852496,1.078384,0.095356\N,0,0.095
133,2.234671,0.146152\N,0,2.158769,1.035738,0.008163\C,0,2.415231,-0.3
09883,-0.069768\N,0,3.69953,-0.780227,-0.118015\H,0,0.702755,3.044532,
0.192251\H,0,-1.645654,1.116693,-0.769963\H,0,3.803318,-1.726543,-0.45
2778\H,0,-0.52006,-2.423465,0.555174\H,0,4.376526,-0.121582,-0.472625\
C,0,-0.997236,-0.673394,0.089255\N,0,-1.338802,-1.856619,0.337544\N,0,
-1.936145,0.332435,-0.194167\C,0,-3.252856,0.051963,-0.165868\N,0,-4.3
97168,-0.124499,-0.148857\\Version=EM64L-G09RevA.01\State=1-A\HF=-594.
0526551\RMSD=3.301e-09\Dipole=2.5882976,1.9560299,-0.2754967\Quadrupol
e=-13.5610921,8.8080533,4.7530389,-9.6224423,-3.5764492,2.1600771\PG=C
01 [X(C4H6N8)]\\@

HD-TS3

1\1\GINC-LOCALHOST\SP\RM062X\Aug-CC-pVTZ\C4H6N8\ROOT\25-Nov-2014\0\\#
m062x/aug-cc-pvtz scrf=(cpcm,solvent=water) geom=check guess=read\\Tit
le Card Required\\0,1\N,0,1.6245363559,0.7721022303,0.405055481\N,0,0.
4355133085,0.1293942957,0.0653479903\H,0,-1.1254426759,-2.081932559,-0
.4263233664\C,0,0.7242371411,-1.1840163987,-0.290295374\N,0,-0.1710111
211,-2.0762165274,-0.7672538162\N,0,2.010007595,-1.3994370174,-0.17307
68356\C,0,2.5048093136,-0.1866865796,0.2384856533\N,0,3.8438371223,-0.
0134342754,0.4232484941\H,0,0.2335985173,-2.9882906951,-0.9272339703\H
,0,-2.0076644628,1.8201027399,-0.3343015152\H,0,4.120172309,0.78623860
84,0.9721654742\H,0,0.1019946734,2.7736926128,-0.0626422136\H,0,4.3715
694043,-0.8595910978,0.5728334442\C,0,-0.6679058094,0.9093049089,-0.11
36492225\N,0,-0.7363786357,2.2025628838,-0.076783052\N,0,-1.9503769562
,0.5007972257,-0.4050825696\C,0,-2.5345440581,-0.53917169,0.1826624944
\N,0,-3.0404542614,-1.4854610953,0.6366954037\\Version=EM64L-G09RevA.0
1\State=1-A\HF=-593.9922136\RMSD=3.299e-09\Dipole=1.8292421,0.5329267,

-0.0953829\Quadrupole=-2.4223899,4.3481217,-1.9257317,-4.6301032,7.377
7715,2.1364302\PG=C01 [X(C4H6N8)]\\@

HD-P

1\1\GINC-LOCALHOST\SP\RM062X\Aug-CC-pVTZ\C4H6N8\ROOT\25-Nov-2014\0\\#
m062x/aug-cc-pvtz scrf=(cpcm,solvent=water) geom=check guess=read\\Tit
le Card Required\\0,1\N,0,1.5105918768,0.7553290823,0.5681639985\N,0,0
.3287294975,0.1166853789,0.1914006143\H,0,-1.1264712785,-2.1334334816,
-0.5237020952\C,0,0.6543796941,-1.1351727031,-0.3117944874\N,0,-0.2016
874145,-1.9908994704,-0.9190050128\N,0,1.950550619,-1.3283750043,-0.22
3964425\C,0,2.4165780164,-0.157945442,0.3039291655\N,0,3.7533313726,0.
0359481032,0.505627193\H,0,0.2647495429,-2.8394399711,-1.2105261296\H,
0,4.0007214899,0.7678337354,1.1542537305\H,0,0.4137051018,2.5334130497
,-0.0575352944\H,0,4.2980264169,-0.8103381715,0.5741321083\C,0,-0.8064
510519,0.9124151576,0.0246166836\N,0,-0.5412856697,2.2147970653,-0.135
5906271\N,0,-2.0441447294,0.508259792,0.008915677\C,0,-2.3901130121,-0
.6925728363,0.4350689506\N,0,-2.7622712373,-1.7407192872,0.787312136\H
,0,-1.3174011044,2.8560790631,-0.0931460459\\Version=EM64L-G09RevA.01\
State=1-A\HF=-594.0715959\RMSD=9.873e-09\Dipole=2.026074,1.6335223,-0.
214462\Quadrupole=-5.3616227,7.9143782,-2.5527555,-6.7954482,7.2550818
,3.4392029\PG=C01 [X(C4H6N8)]\\@

1-amino-1,2,3-triazolium

1\1\GINC-LOCALHOST\SP\RM062X\Aug-CC-pVTZ\C2H5N4(1+)\ROOT\18-Nov-2014\0 \\#
m062x/aug-cc-pvtz scrf=(cpcm,solvent=water)\\Title Card Required\\
1,1\C,0,-0.167115,1.136387,-0.000113\N,0,0.627026,0.03386,0.000082\H,0
,-2.011047,-1.421193,-0.000297\H,0,-2.411381,1.129601,-0.000281\H,0,0.
24579,2.133723,-0.000182\N,0,-0.044367,-1.086864,-0.000021\C,0,-1.4466
8,0.645207,-0.00017\N,0,-1.28996,-0.702961,-0.000182\N,0,2.012599,0.07
6191,0.000208\H,0,2.36106,-0.385419,0.837818\H,0,2.361255,-0.387859,-0
.835966\\Version=EM64L-G09RevA.01\State=1-A\HF=-297.9861395\RMSD=2.670
e-09\Dipole=-0.9451163,-0.0416858,0.0007822\Quadrupole=8.0454004,-2.11
14833,-5.9339171,-0.5785661,0.0064382,0.0026305\PG=C01 [X(C2H5N4)]\\@

1-amino-1,2,3-triazole

1\1\GINC-LOCALHOST\SP\RM062X\Aug-CC-pVTZ\C2H4N4\ZHANGZH\18-Nov-2011\0\ \#
m062x/aug-cc-pvtz scrf=(cpcm,solvent=water)\\Title Card Required\\0
,1\C,0,-0.252495,1.113092,-0.000047\N,0,0.592451,0.059133,-0.000024\H,
0,-2.473549,0.976286,0.000052\H,0,0.108273,2.128262,-0.000119\N,0,-0.0
67061,-1.103295,-0.000012\C,0,-1.494465,0.524632,0.000022\N,0,-1.33235
3,-0.827531,0.000032\N,0,1.980054,0.127909,-0.000033\H,0,2.317703,-0.3
61739,0.824622\H,0,2.317699,-0.362659,-0.824142\\Version=EM64L-G09RevA
.01\State=1-A\HF=-297.5566064\RMSD=3.985e-09\Dipole=1.2142405,1.262135
,0.0003445\Quadrupole=2.5961165,-2.4389786,-0.1571379,-5.5365356,0.001

7307,0.0009721\PG=C01 [X(C2H4N4)]\\@

AD-1

1\1\GINC-LOCALHOST\SP\RM062X\Aug-CC-pVTZ\C4H5N7\ROOT\14-Nov-2014\0\\# m062x/aug-cc-pvtz scrf=(cpcm,solvent=water)\\Title Card Required\\0,1\ C,0,-1.060226,-1.652741,0.822303\C,0,-1.973545,-0.629569,0.815667\N,0, -0.632882,-0.377755,-0.931792\H,0,-0.891451,-2.467686,1.50672\H,0,-2.7 82355,-0.34634,1.468197\N,0,-0.29227,-1.449225,-0.27429\N,0,-1.65401,0 .104336,-0.277201\N,0,-2.214474,1.335479,-0.589138\H,0,-2.297365,1.383 974,-1.601602\C,0,1.340493,1.517996,0.391651\N,0,0.334195,2.096562,0.5 56217\N,0,2.489052,0.903281,0.219815\C,0,2.407246,-0.348839,-0.123817\ N,0,2.328149,-1.483693,-0.426846\H,0,-1.511491,2.020913,-0.266767\H,0, 0.694539,-1.814845,-0.478733\\Version=EM64L-G09RevA.01\State=1-A\HF=-5 38.5910599\RMSD=2.898e-09\Dipole=-3.6297953,-1.6884077,0.2240861\Quadr upole=-3.6446256,-0.9274376,4.5720632,3.2134337,0.1584555,-7.4640003\P G=C01 [X(C4H5N7)]\\@

AD-TS1

1\1\GINC-LOCALHOST\SP\RM062X\Aug-CC-pVTZ\C4H5N7\ROOT\14-Nov-2014\0\\# m062x/aug-cc-pvtz scrf=(cpcm,solvent=water)\\Title Card Required\\0,1\ C,0,2.197027,-0.231827,0.837782\C,0,1.047902,-0.939967,1.049941\N,0,1. 162661,-0.473475,-1.124036\H,0,2.955427,0.125532,1.514386\H,0,0.583557 ,-1.351641,1.929045\N,0,2.226045,0.003755,-0.488321\N,0,0.450961,-1.04 0625,-0.181758\N,0,-0.770729,-1.546363,-0.369626\H,0,-0.884417,-1.5913 61,-1.384597\C,0,-2.06338,0.081785,0.010533\N,0,-3.06919,-0.445569,0.4 85277\N,0,-1.504911,1.117662,-0.462398\C,0,-0.434953,1.742228,-0.01487 3\N,0,0.509174,2.353402,0.298989\H,0,-3.032276,-1.45768,0.544878\H,0,2 .870068,0.580342,-1.010887\\Version=EM64L-G09RevA.01\State=1-A\HF=-538 .5477162\RMSD=4.389e-09\Dipole=2.7007332,-1.8678127,0.294089\Quadrupol e=6.6258461,-9.5558739,2.9300278,2.4945556,1.5645097,-2.527754\PG=C01 [X(C4H5N7)]\\@

AD-2

1\1\GINC-LOCALHOST\SP\RM062X\Aug-CC-pVTZ\C4H5N7\ROOT\14-Nov-2014\0\\# m062x/aug-cc-pvtz scrf=(cpcm,solvent=water)\\Title Card Required\\0,1\ C,0,2.192024,-0.186791,0.922011\C,0,0.897888,-0.574384,1.143406\N,0,1. 280145,-0.684723,-1.044254\H,0,2.963718,0.161779,1.587752\H,0,0.279262 ,-0.62603,2.024564\N,0,2.362254,-0.30185,-0.413389\N,0,0.407192,-0.841 919,-0.09492\N,0,-0.900496,-1.166498,-0.35354\H,0,-0.968197,-1.391834, -1.344565\C,0,-1.936745,-0.103469,0.044787\N,0,-3.133978,-0.536043,0.0 7938\N,0,-1.519258,1.1224,0.36943\C,0,-0.429874,1.67292,-0.102212\N,0, 0.558093,2.209117,-0.444054\H,0,-3.161363,-1.546949,-0.035856\H,0,3.16 9152,-0.050026,-0.970416\\Version=EM64L-G09RevA.01\State=1-A\HF=-538.5 816148\RMSD=3.765e-09\Dipole=4.5466022,-3.2898066,0.3276612\Quadrupole

=2.7188439,-8.4986577,5.7798138,1.7639797,4.5972659,2.2101708\PG=C01 [
X(C4H5N7)]\\@

AD-TS2

1|1| GINC-LOCALHOST |SP|RM062X|Aug-CC-pVTZ|C4H5N7|ZHANGZH|22-Feb-2015|0|| #
m062x/aug-cc-pvtz scrf=(cpcm,solvent=water)||Title Card Required||0,
1|C,0,2.236996,0.100053,0.988913|C,0,0.891571,-0.129771,1.039086|N,0,1
.597361,-0.724088,-0.982935|H,0,2.927132,0.509509,1.707923|H,0,0.14249
3,0.044092,1.795698|N,0,2.603602,-0.294731,-0.252296|N,0,0.567735,-0.6
30087,-0.193015|N,0,-0.660104,-1.001213,-0.664455|H,0,-1.381317,-1.988
727,-0.291498|C,0,-1.762813,-0.382575,0.01392|N,0,-2.488191,-1.448748,
0.287257|N,0,-1.953084,0.895773,0.267426|C,0,-1.02899,1.738622,-0.1762
29|N,0,-0.204183,2.50194,-0.49687|H,0,3.519283,-0.268158,-0.680717|H,0
,-3.470125,-1.346616,0.518661||Version=IA32W-G09RevA.02|State=1-A|HF=-
538.5419139|RMSD=3.177e-009|Dipole=3.6745495,-2.0018103,1.656681|Quadr
upole=16.9264198,-15.2738314,-1.6525884,1.5721143,2.0619439,1.9102578|
PG=C01 [X(C4H5N7)]||@

AD-3

1\1\GINC-LOCALHOST\SP\RM062X\Aug-CC-pVTZ\C4H5N7\ROOT\25-Nov-2014\0\\#
m062x/aug-cc-pvtz scrf=(cpcm,solvent=water) geom=check guess=read\\Tit
le Card Required\\0,1\C,2.3374444669,-0.2726138786,0.8427596414\C,1.12
14320239,-0.6457016474,1.3431547345\N,0.9402391668,-0.5714957734,-0.86
56831094\H,3.268372231,-0.0102005337,1.3169902526\H,0.7402702336,-0.77
87239262,2.3409209532\N,2.1583040584,-0.2507978854,-0.4974283718\N,0.3
123714425,-0.7885405434,0.2567831952\N,-0.9978060416,-1.1604095852,0.2
930305188\C,-1.8423658537,-0.146783141,-0.0018426165\N,-3.1451218228,-
0.549343273,-0.0433566194\N,-1.6438039935,1.1484161903,-0.2111652232\C
,-0.4752149288,1.7380789554,-0.06768649\N,0.5265835696,2.3365950695,0.
0305650073\H,2.8064890399,0.048055393,-1.2138593071\H,-3.8019740004,0.
1179242815,-0.4142294008\H,-3.3239452119,-1.5317484418,-0.1744834448\\
Version=EM64L-G09RevA.01\State=1-A\HF=-538.6178698\RMSD=5.907e-09\Dipo
le=2.1887961,-2.2403878,0.3539333\Quadrupole=17.606725,-17.1495024,-0.
4572226,-1.1490918,2.1499765,-0.2780244\PG=C01 [X(C4H5N7)]\\@

AD-TS3

1\1\GINC-LOCALHOST\SP\RM062X\Aug-CC-pVTZ\C4H5N7\ROOT\14-Nov-2014\0\\#
m062x/aug-cc-pvtz scrf=(cpcm,solvent=water)\\Title Card Required\\0,1\
C,0,1.754621,-1.536985,-0.539089\C,0,2.362381,-0.305258,-0.554095\N,0,
0.547943,-0.191835,0.707506\H,0,2.005297,-2.462503,-1.030966\H,0,3.238
169,0.0924,-1.039514\N,0,0.659088,-1.403594,0.248687\N,0,1.570004,0.47
0071,0.225027\N,0,1.71489,1.840086,0.413083\H,0,1.781834,2.009839,1.41
365\C,0,-1.854871,1.280197,-0.316744\N,0,-1.107195,2.148329,-0.548783\
N,0,-2.713032,0.318596,-0.029521\C,0,-2.218786,-0.86346,0.072426\N,0,-

1.773705,-1.953829,0.173277\H,0,0.843475,2.264154,0.060133\H,0,-0.4147
99,-1.945615,0.296785\\Version=EM64L-G09RevA.01\State=1-A\HF=-538.5846
917\RMSD=9.575e-09\Dipole=3.637944,-1.0663535,0.0926261\Quadrupole=1.2
368939,-3.5395905,2.3026966,-2.4161939,-1.4049571,6.0821987\PG=C01 [X(
C4H5N7)]\\@

AD-TS4

1\1\GINC-LOCALHOST\SP\RM062X\Aug-CC-pVTZ\C4H5N7\ROOT\24-Nov-2014\0\\#
m062x/aug-cc-pvtz scrf=(cpcm,solvent=water)\\Title Card Required\\0,1\
C,0,-2.466091,1.014705,-0.386031\C,0,-1.170918,1.119897,0.051952\N,0,-
1.8866,-1.009695,0.122951\H,0,-3.132151,1.7861,-0.737345\H,0,-0.468458
,1.933533,0.162424\N,0,-2.854814,-0.293098,-0.326007\N,0,-0.861274,-0.
17027,0.354234\N,0,0.313668,-0.727948,0.84575\H,0,0.382561,-1.94263,0.
676375\C,0,2.017587,1.280343,-0.150069\N,0,1.862107,2.358346,0.267301\
N,0,2.207382,0.057109,-0.623589\C,0,1.47503,-0.901149,-0.146633\N,0,1.
422296,-2.196251,-0.244353\H,0,0.572004,-0.313659,1.746222\H,0,2.09304
9,-2.673466,-0.836999\\Version=EM64L-G09RevA.01\State=1-A\HF=-538.5391
682\RMSD=9.465e-09\Dipole=0.0914951,-0.9368118,1.0117375\Quadrupole=-6
.3064135,-1.0254635,7.331877,-14.9323013,0.4980936,-1.7890356\PG=C01 [
X(C4H5N7)]\\@

AD-P

1\1\GINC-LOCALHOST\SP\RM062X\Aug-CC-pVTZ\C4H5N7\ROOT\24-Nov-2014\0\\#
m062x/aug-cc-pvtz scrf=(cpcm,solvent=water) geom=check guess=read\\Tit
le Card Required\\0,1\C,0,-2.8655425033,0.3088346713,-0.3813574838\C,0
,-1.6478512526,0.9172206562,-0.5408503576\N,0,-1.4928298998,-0.8094341
018,0.872379218\H,0,-3.819863913,0.546404735,-0.8230951686\H,0,-1.2854
948707,1.7547522269,-1.1147553134\N,0,-2.7193486587,-0.7399463131,0.48
13676891\N,0,-0.8294371107,0.190370989,0.2609595765\N,0,0.5108713044,0
.3769435901,0.4452925674\C,0,3.0945731295,0.5937132271,0.9078116843\N,
0,3.4532663783,1.4941725609,1.5530223274\N,0,2.6941629553,-0.452692921
1,0.1841976335\C,0,1.417613579,-0.5493389724,-0.0418415383\N,0,0.93667
07719,-1.5252213279,-0.8411332721\H,0,0.7809028053,0.907439665,1.26837
07611\H,0,1.6275057499,-2.2026722385,-1.1306162781\H,0,-0.0051502448,-
1.8621625867,-0.6967026553\\Version=EM64L-G09RevA.01\State=1-A\HF=-538
.6313304\RMSD=7.477e-09\Dipole=-1.8940759,-0.3787565,-2.0091446\Quadru
pole=-11.8556591,7.0537908,4.8018683,-14.0937497,-1.3956916,-0.4961211
\PG=C01 [X(C4H5N7)]\\@

Figure S3. Diamond Representation of the Molecular Structure of **4**. Displacement Ellipsoids are Shown at 50% Probability Level. (b) Unit Cell Packing of **4**.

a)
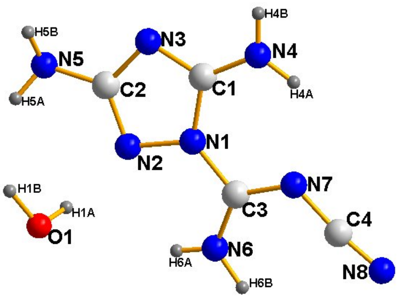

b)
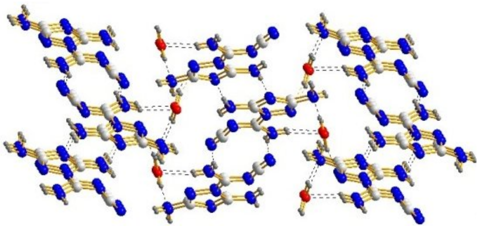

Figure S4. Diamond Representation of the Molecular Structure of **11**. Displacement Ellipsoids are Shown at 50% Probability Level. (b) Unit Cell Packing of **11**.

Spectrometric data

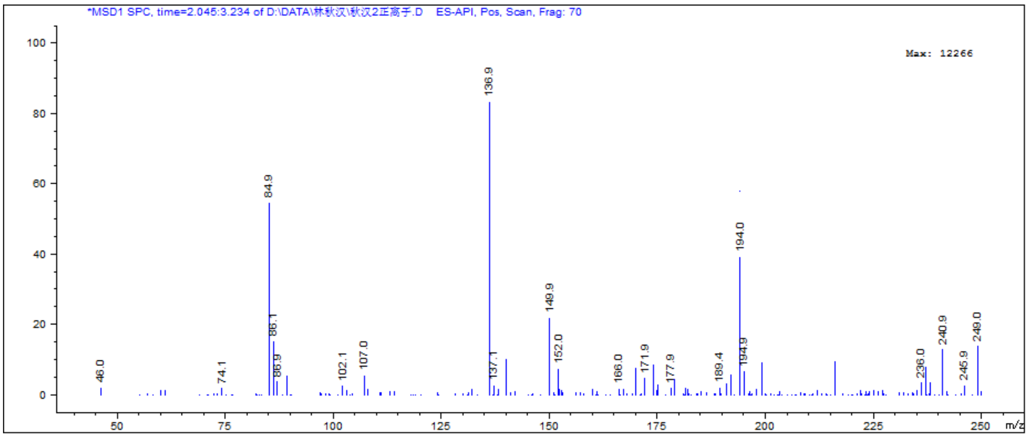

Figure S5. MS spectrum of **4**.

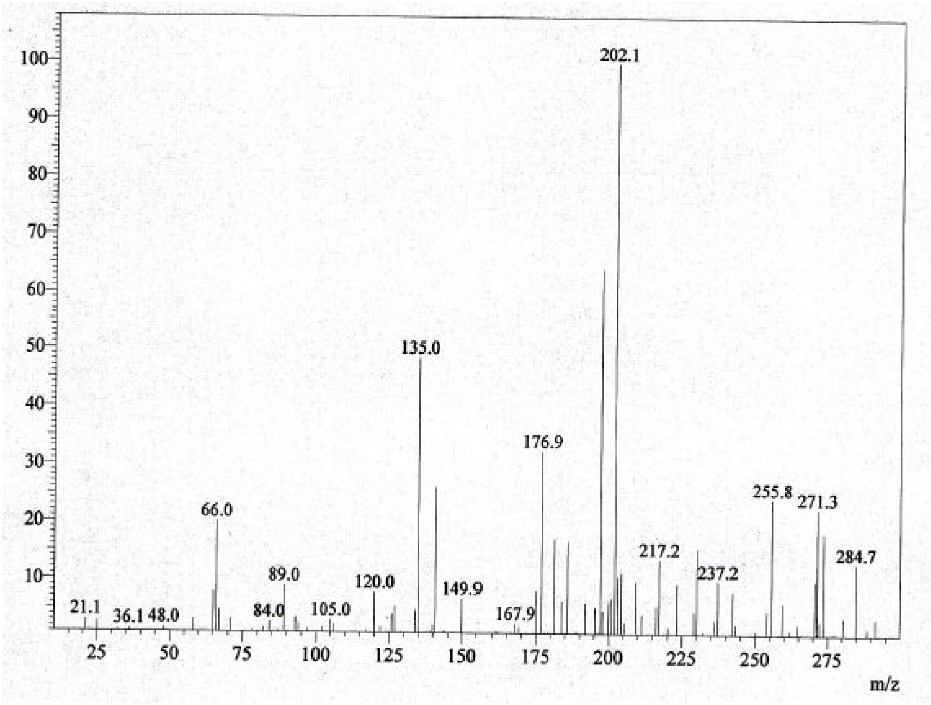

Figure S6. MS spectrum of **5**.

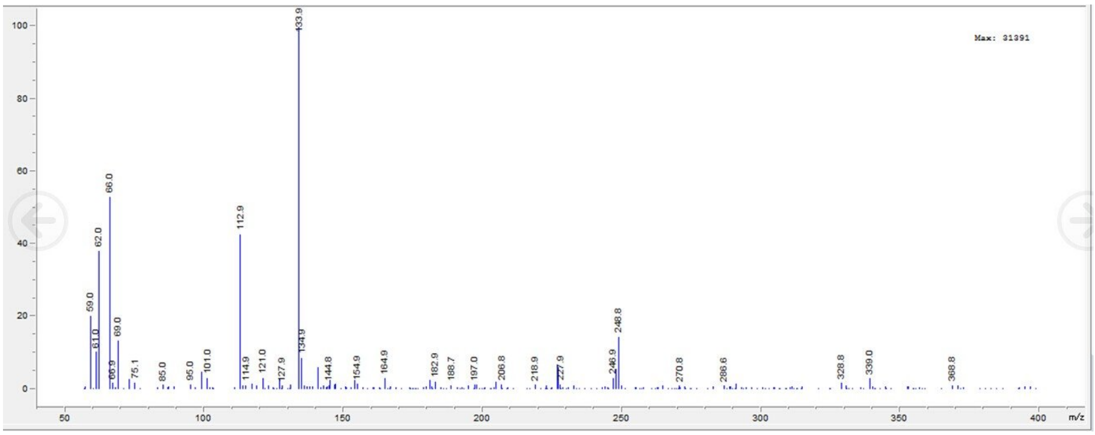

Figure S7. MS spectrum of **6**.

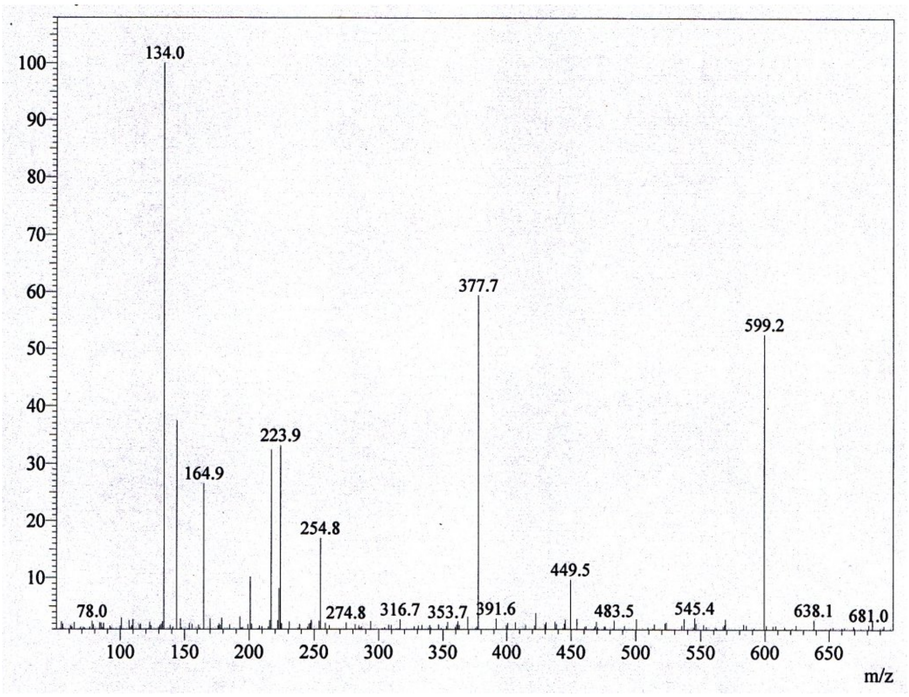

Figure S8. MS spectrum of **7**.

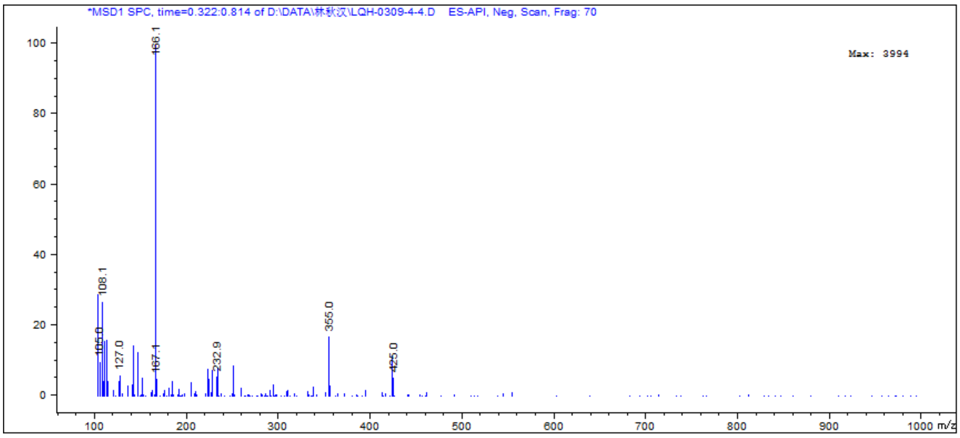

Figure S9. MS spectrum of **8**.

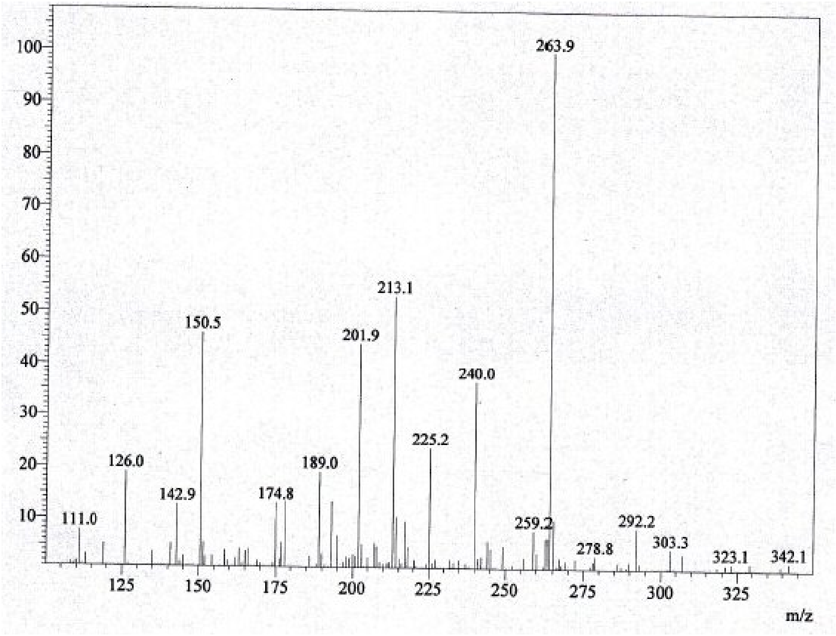

Figure S10. MS spectrum of **9**.

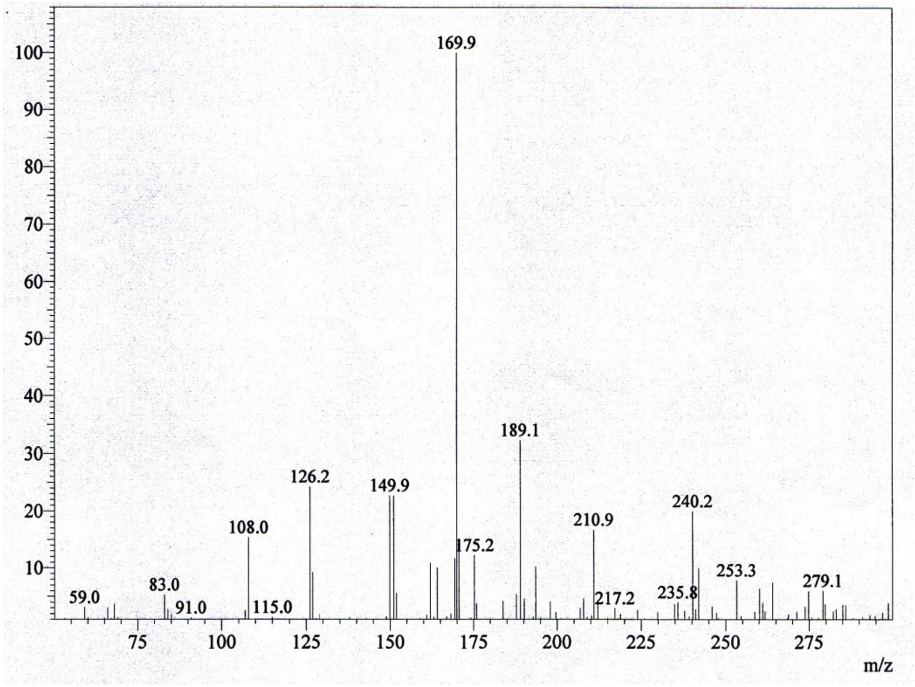

Figure S11. MS spectrum of **10**.

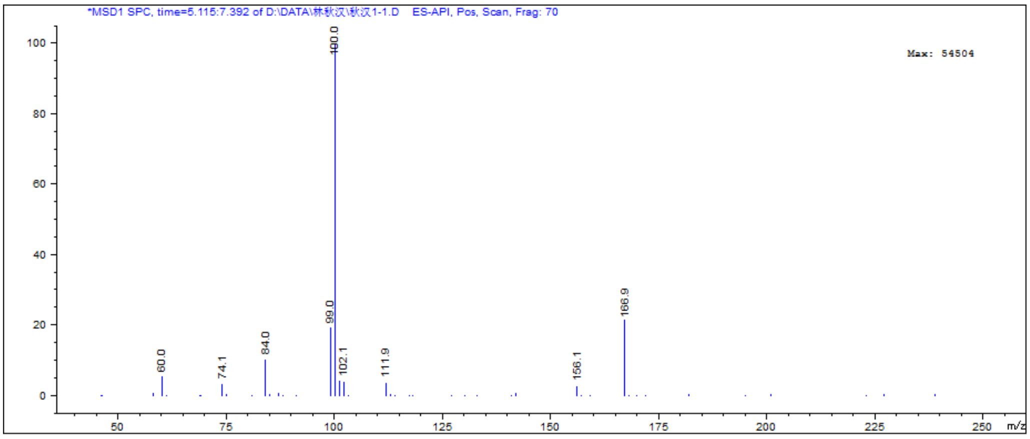

Figure S12. MS spectrum of **11**.

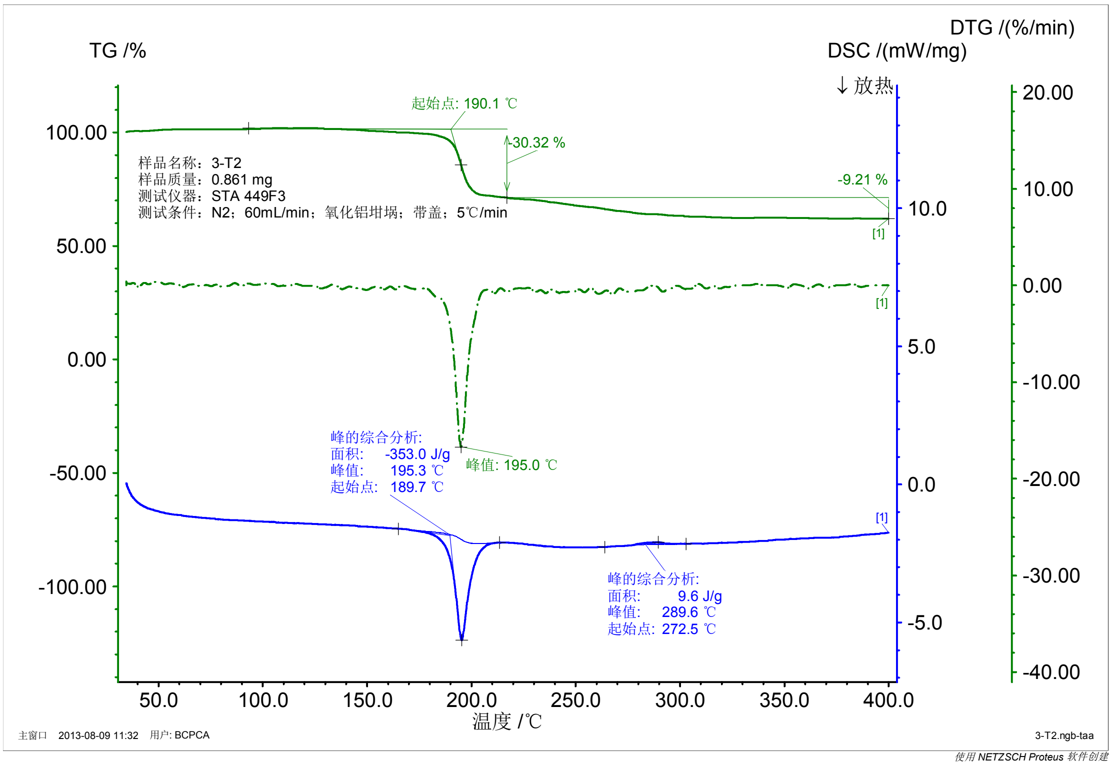

Figure S13. DSC thermogram of 4 (heating rate of 5°C·min^{-1}).

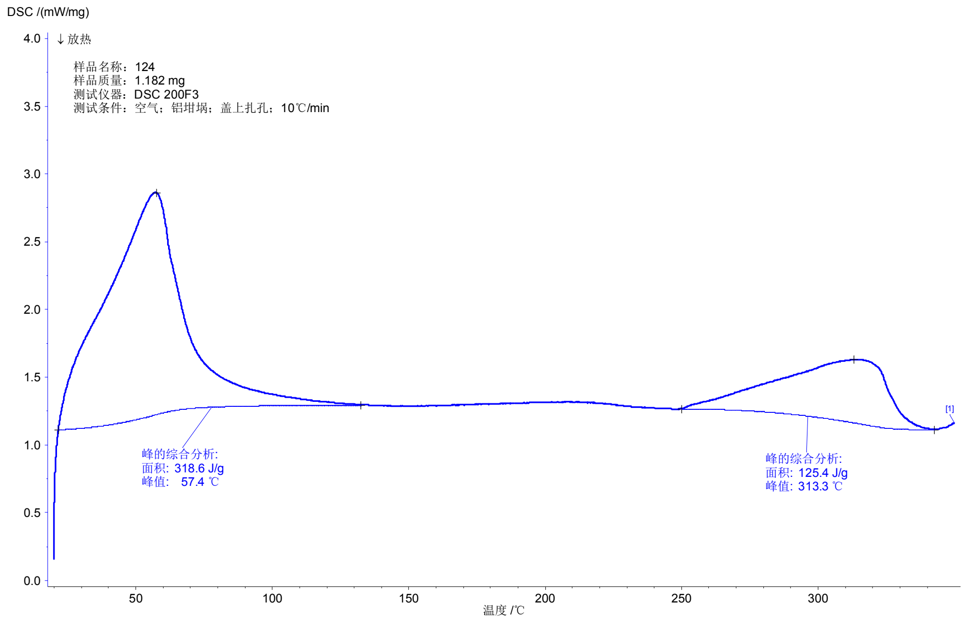

Figure S14. DSC thermogram of **5** (heating rate of 10°C·min^{-1}).

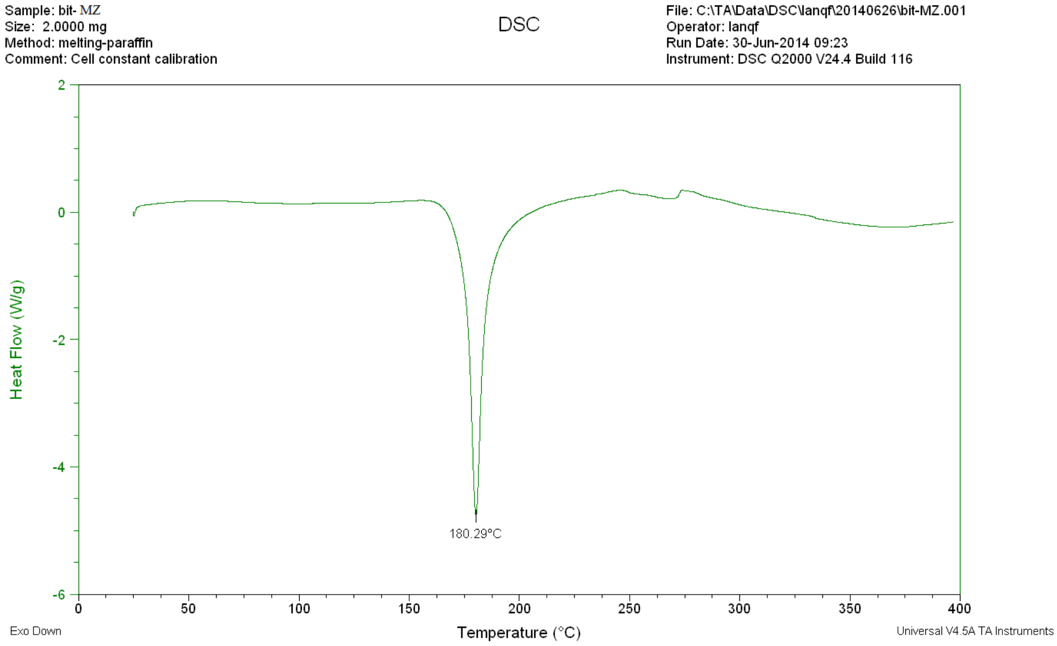

Figure S15. DSC thermogram of **6** (heating rate of 10°C·min^{-1}).

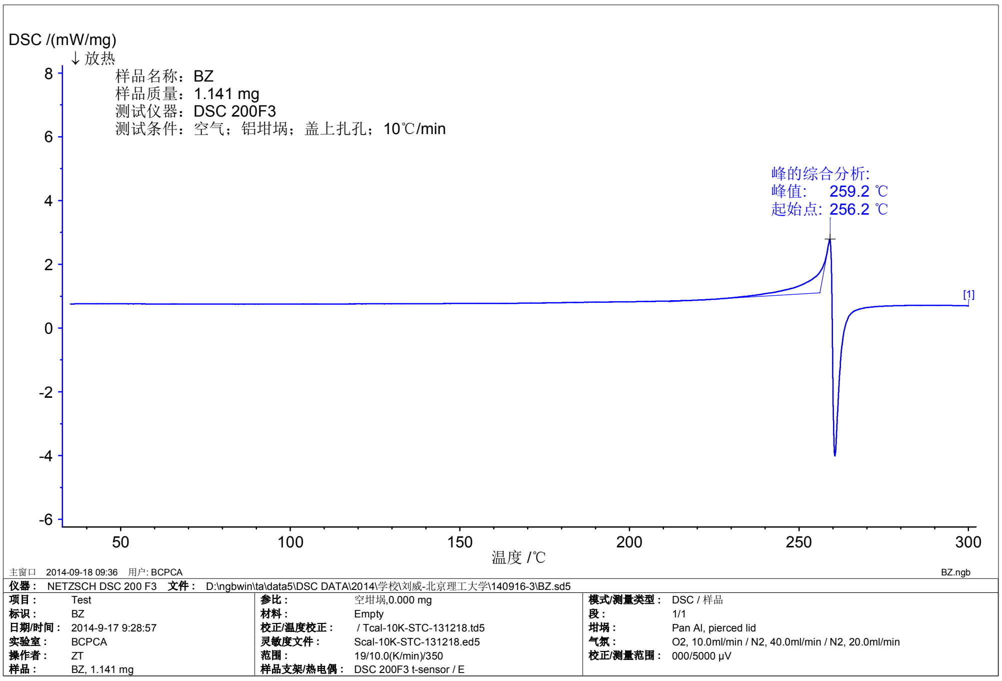

Figure S16. DSC thermogram of **7** (heating rate of 10°C·min^{-1}).

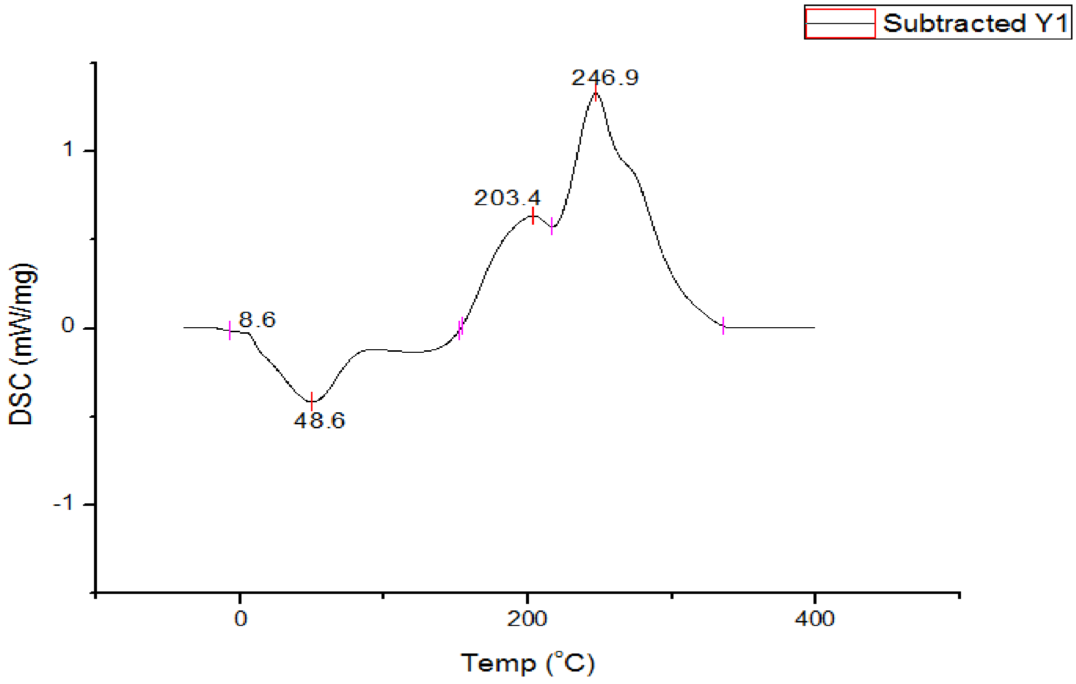

Figure S17. DSC thermogram of **8** (heating rate of 5°C·min^{-1}).

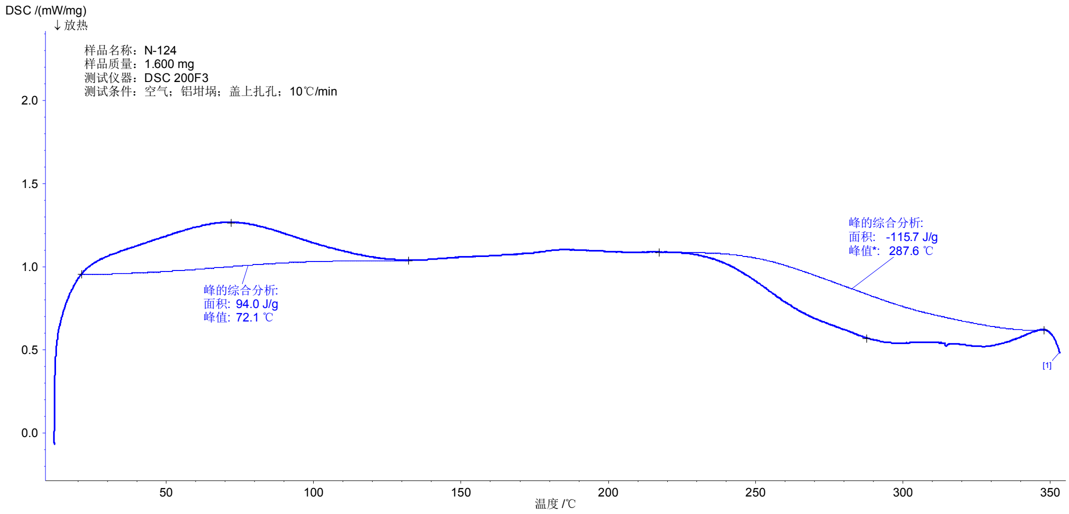

Figure S18. DSC thermogram of **9** (heating rate of 10°C·min^{-1}).

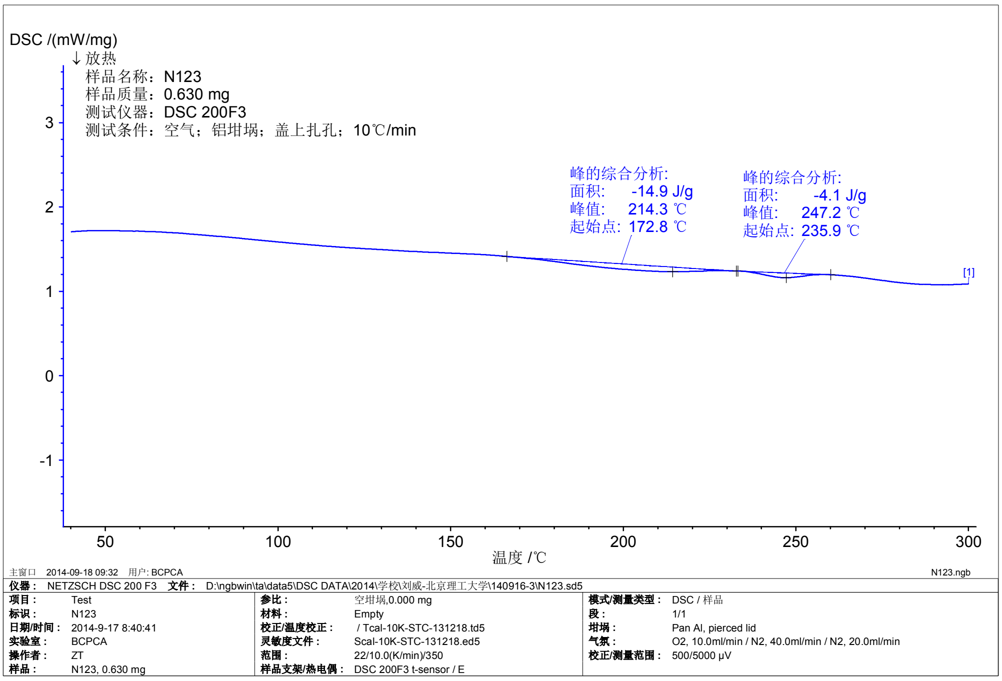

Figure S19. DSC thermogram of **10** (heating rate of 10°C·min^{-1}).

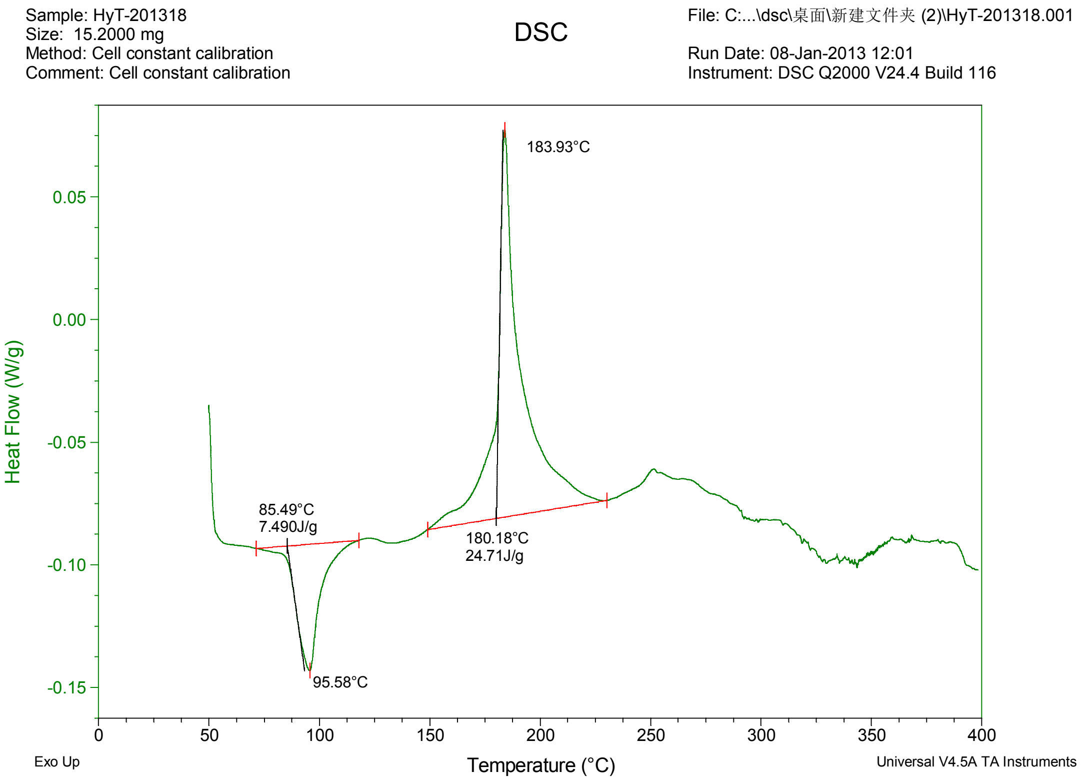

Figure S20. DSC thermogram of **11** (heating rate of 5°C·min^{-1}).

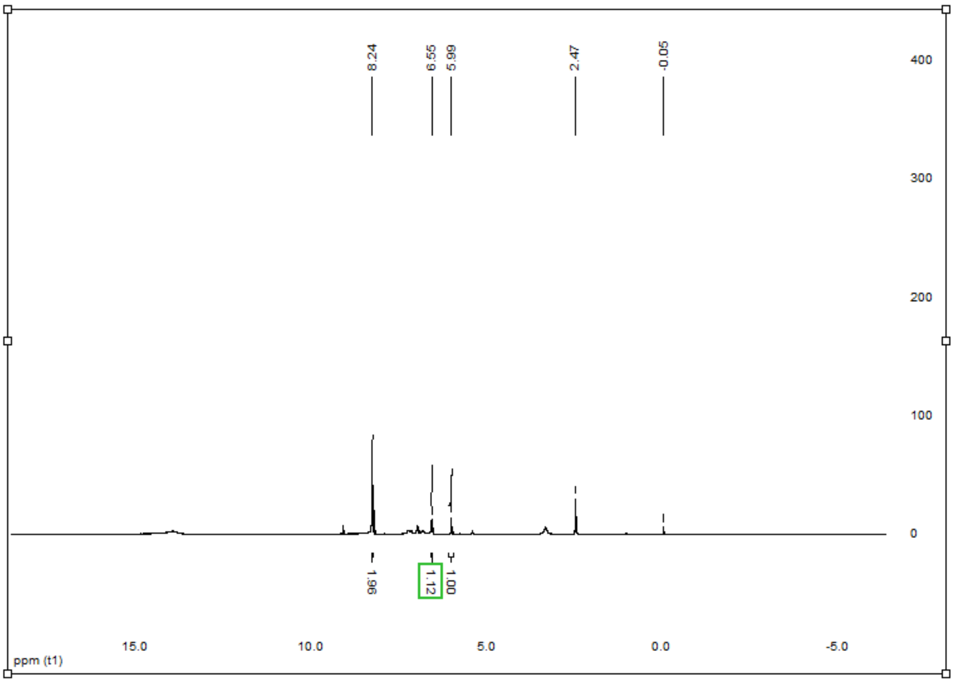

Figure S21. ^{1}H NMR spectrum of **5** in DMSO-d6.

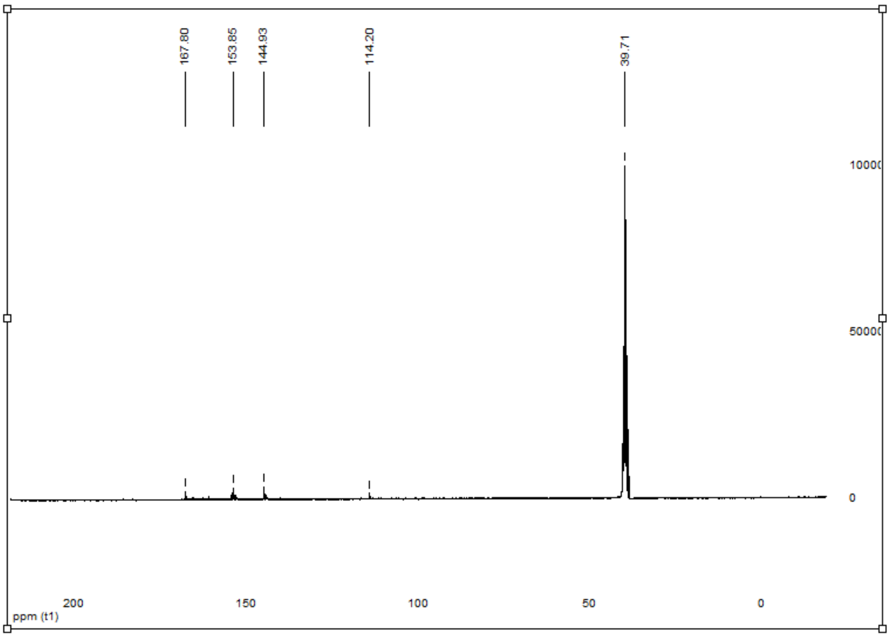

Figure S22. ^{13}C NMR spectrum of **5** in DMSO-d6.

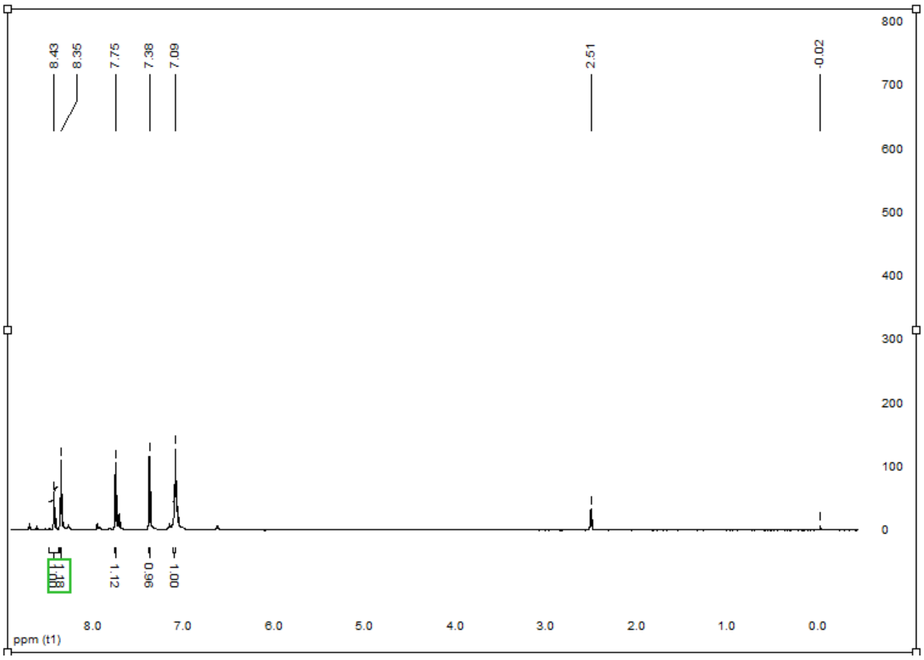

Figure S23. ^{1}H NMR spectrum of **6** in DMSO-d6.

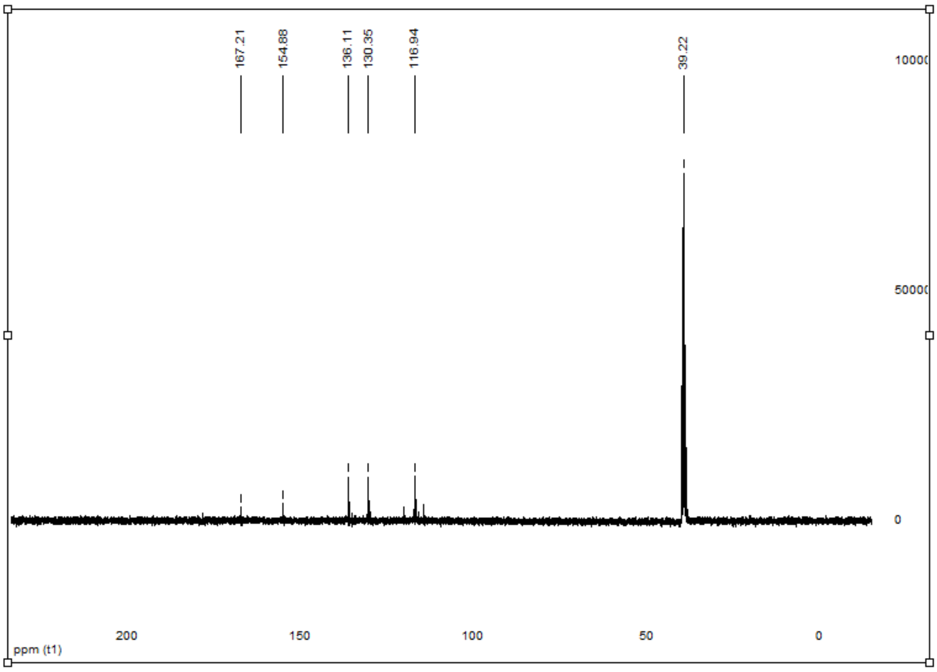

Figure S24. ^{13}C NMR spectrum of **6** in DMSO-d6.

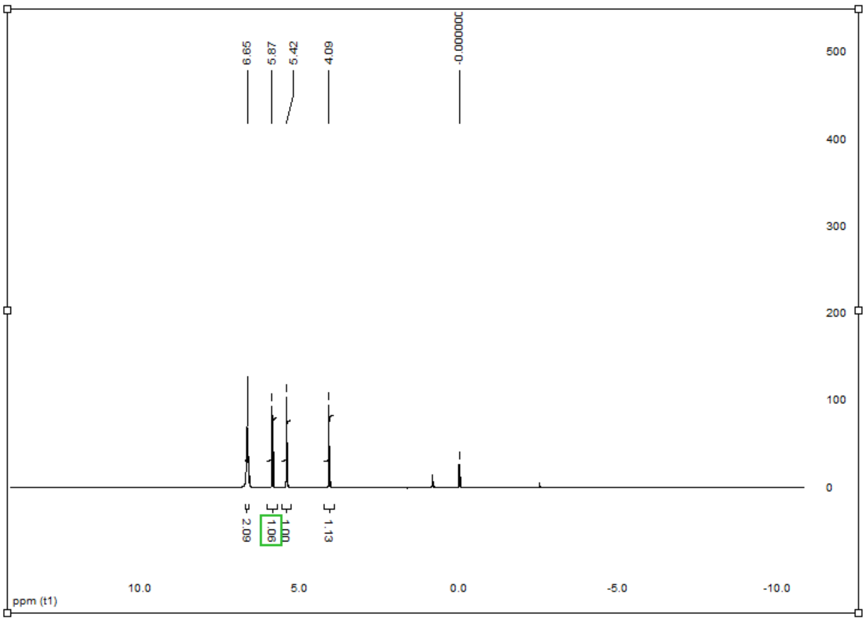

Figure S25. ^{1}H NMR spectrum of **7** in DMSO-d6.

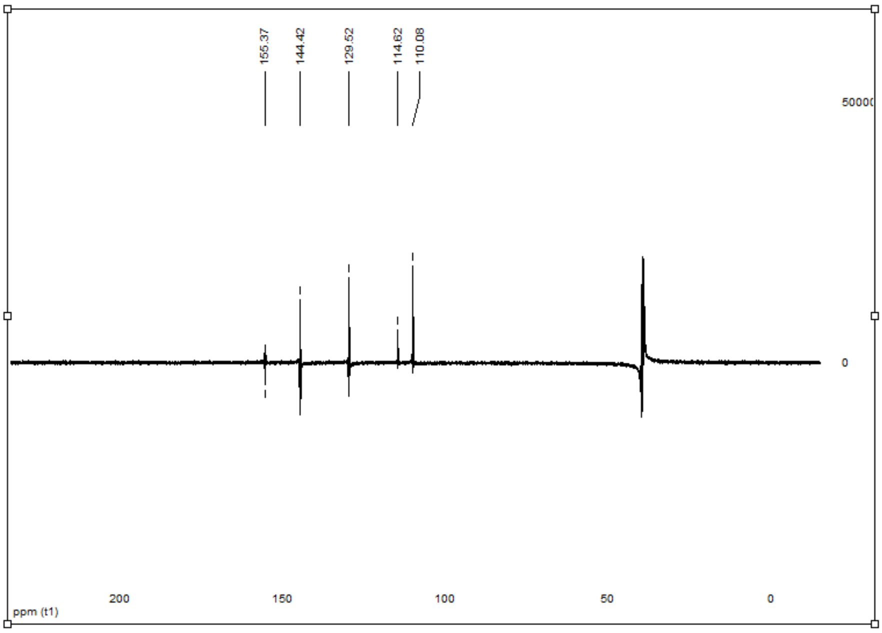

Figure S26. ^{13}C NMR spectrum of **7** in DMSO-d6.

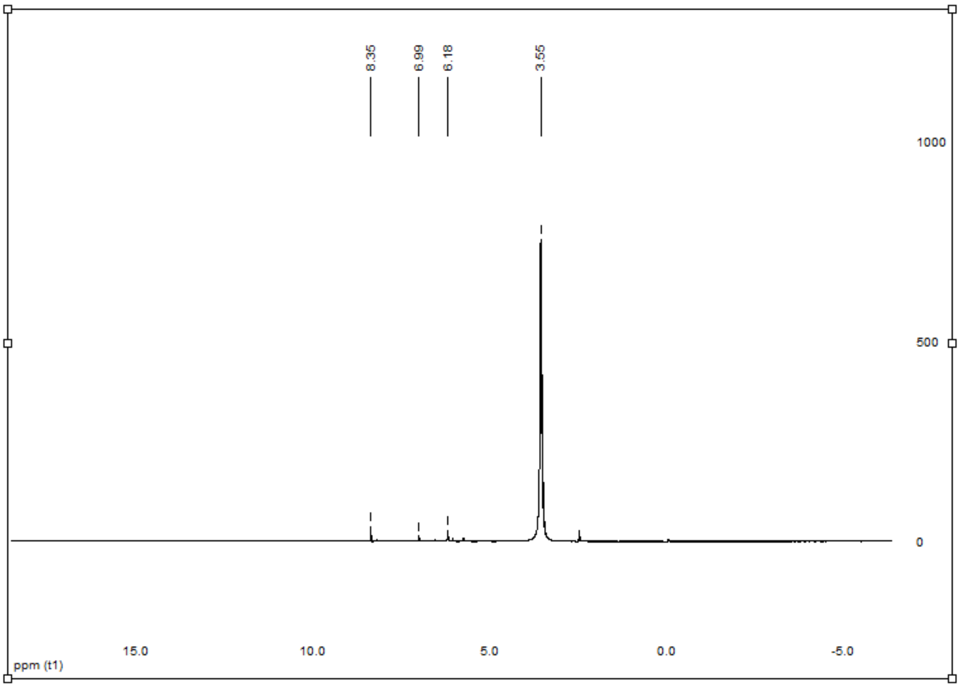

Figure S27. ^{1}H NMR spectrum of **9** in DMSO-d6.

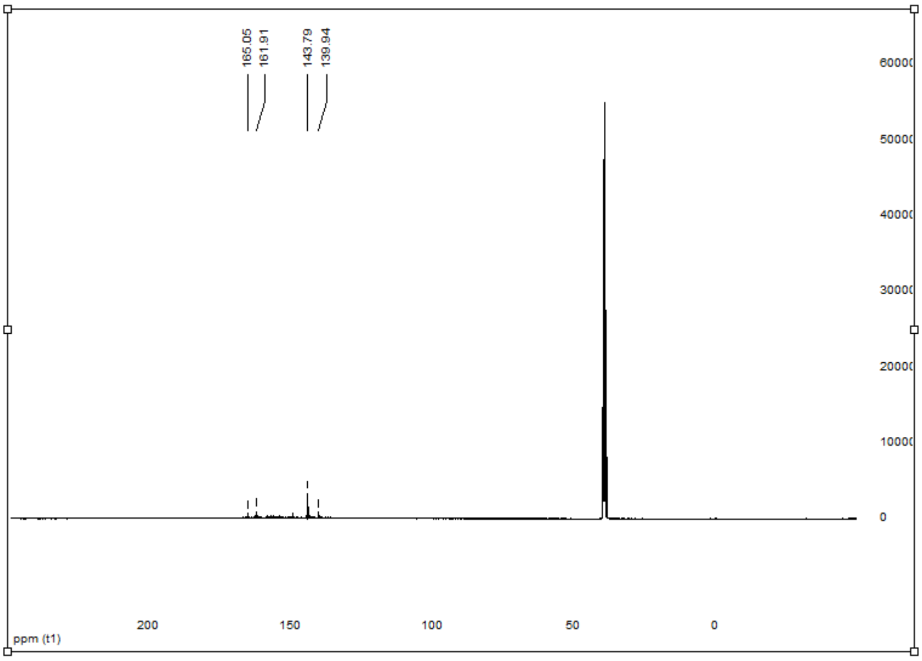

Figure S28. ^{13}C NMR spectrum of **9** in DMSO-d6.

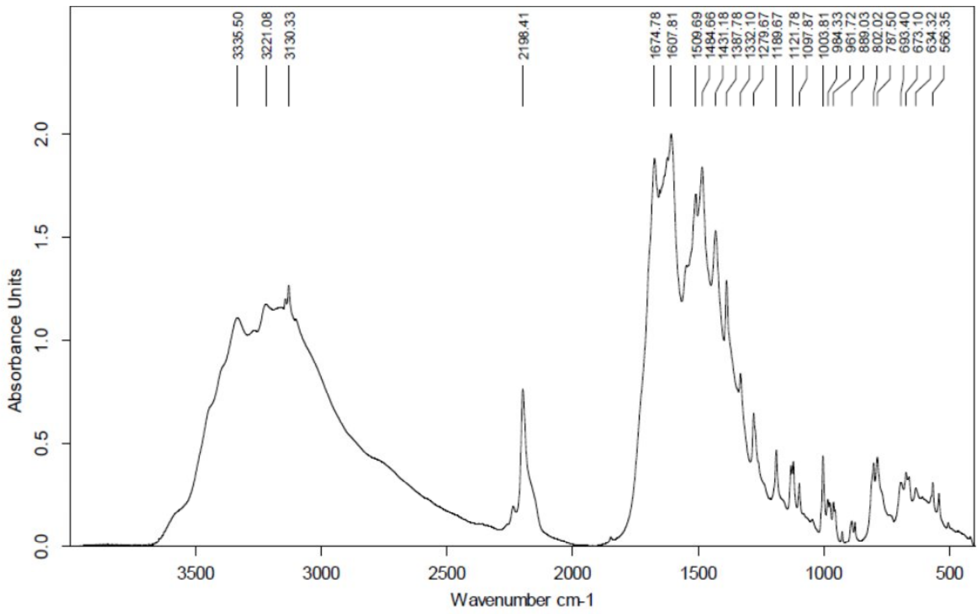

Figure S29. IR spectrum of **5**.

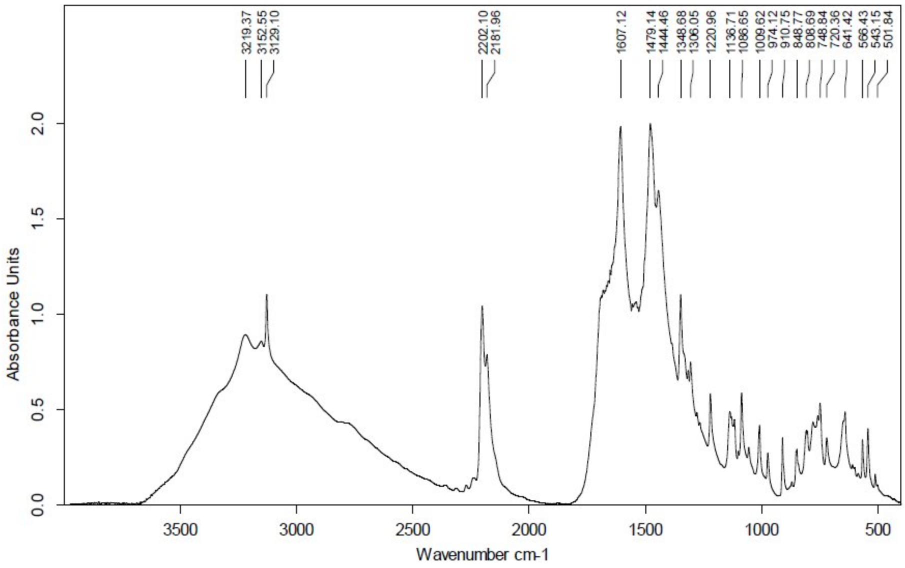

Figure S30. IR spectrum of **6**.

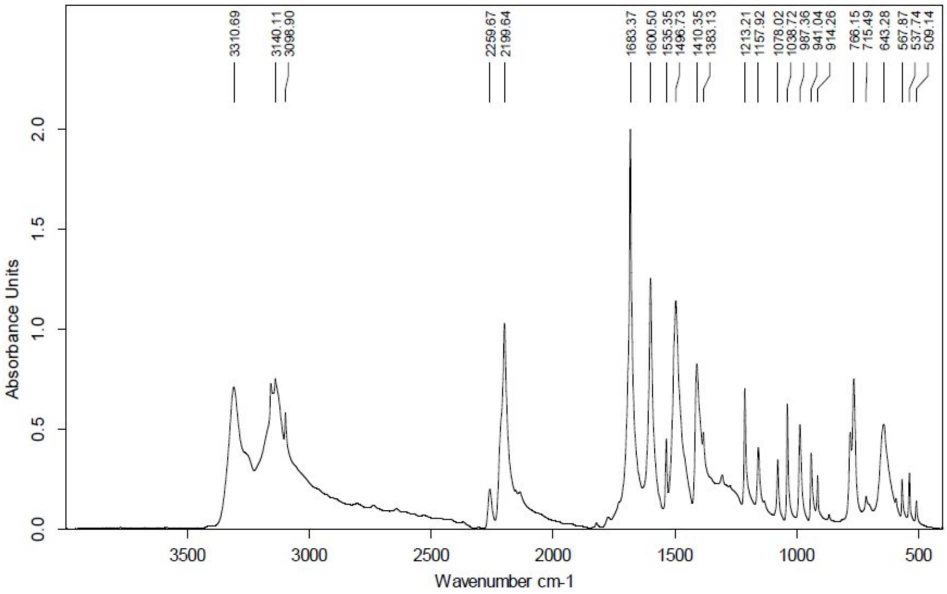

Figure S31. IR spectrum of **7**.

References:

[1] M. Frisch, G. W. Trucks, H. B. Schlegel, G. E. Scuseria, M. A. Robb, J. R. Cheeseman, G. Scalmani, V. Barone, B. Mennucci, G. A. Petersson, *Inc., Wallingford, CT* **2009**, *270*, 271.

[2] W. J. Hehre, L. Radom, P. V. R. Schleyer, J. A. Pople, *Ab initio molecular orbital theory, Vol. 33*, Wiley New York et al., **1986**.

[3] M. Sućeska, *Zagreb, Croatia* **2011**.

[4] Y. Guo, et al. *Chem. Eur. J.*, 2010, 16,3753-3762.

[5] Q. H. Lin, et al. *J. Mater. Chem.*, 2012, 22, 666-674.

[6] Pedley J B. Thermochemical Data and Structure of Organic Compounds, Thermodynamic Research Center, College Station, 1994.

[7] L. X. Liang, K. Wang, C. M. Bian, L. M. Ling and Z. M. Zhou, Chem. Eur. J., 2013, 19, 14902-14910.
[8] K. Fukui, *Accounts of chemical research* **1981**, *14*, 363.
[9] Y. Zhao, D. G. Truhlar, *Theoretical Chemistry Accounts* **2008**, *120*, 215.
[10] C. Qi, R. Zhang, S. Pang, *RSC Advances* **2013**, *3*, 17741.
